# Longitudinal spatial neutrophil profiling during ACT in murine melanoma reveals distinct lymph node infiltration patterns

**DOI:** 10.1038/s41540-026-00765-5

**Published:** 2026-06-11

**Authors:** Gemma van der Voort, Maike Effern, Michelle C. R. Yong, Lukas Kiwitz, Roberta Turiello, Sonia Leonardelli, Susanna Ng, Dillon Corvino, Tobias Bald, Nicole Glodde, Kevin Thurley, Jan Hasenauer, Michael Hölzel

**Affiliations:** 1 Institute for Experimental Oncology, University Hospital Bonn, Bonn, Germany; 2 Life and Medical Sciences (LIMES) Institute, University of Bonn, Bonn, Germany; 3 Bonn Center for Mathematical Life Sciences, University of Bonn, Bonn, Germany

**Keywords:** Cancer, Computational biology and bioinformatics, Immunology

## Abstract

Reactive neutrophil infiltration can restrain CD8^+^ T cell expansion in lymph nodes during adoptive T cell therapy (ACT), yet its spatiotemporal regulation remains incompletely understood. Levaraging flow cytometry and multiplex immunofluorescence data, we performed a time-resolved quantitative assessment of immune cell dynamics in tumor-draining lymph node (tdLN) and non-tumor-draining lymph node (non-tdLN) in a melanoma mouse model receiving ACT. Transferred tumor-reactive CD8^+^ T cells accumulated and expanded early after treatment initiation, showing the highest frequency of a favorable central memory 13 CD8^+^ T cell phenotype in the tdLN. Enhancing innate immune signaling in melanomas increased neutrophil influx into lymph nodes, particularly the non-tdLN; however, within the tdLN, neutrophils were enriched in the T cell zone, which also contained the largest absolute reservoir of transferred CD8^+^ T cells. Together, these findings indicate that tdLN and non-tdLN differ in early neutrophil dynamics and compartmentalization during ACT, influenced by the strength of innate immune signaling in the tumor.

## Introduction

Adoptive T cell therapy (ACT) is a promising strategy for cancer treatment not only in hematological malignancies, but progressively also in solid tumors^1^ such as melanoma^2,3^. Among the different ACT strategies, autologous, ex vivo expanded tumor-infiltrating lymphocytes (TILs) have shown promise for melanoma patients. In a recent phase III clinical trial among patients with advanced melanoma refractory to anti-PD-1 therapy, median progression-free survival increased from 3.1 months in the group receiving ipilimumab immune-checkpoint inhibition to 7.2 months in the TIL group^4^.

Among the TILs, the CD8^+^ T cells are the primary effector cells mediating therapeutic efficacy, and their abundance is predictive of improved treatment outcomes in a variety of cancer types and treatment regimens^5^. For a sustained CD8^+^ T cell response, maintaining a long-term proliferative capacity is essential^6,7^. The tdLN serves as a reservoir of central-memory CD8^+^ T cells (CD44^+^CD62L^+^) with strong self-renewal capacity^8^. It is an essential site for anti-tumor immune coordination and surveillance^9^.

The tdLN undergoes significant changes during tumor progression. In its role as the primary site of antigen presentation and T cell activation, this lymph node is exposed to an influx of antigen-presenting cells and undergoes remodeling of its B and T cell compartments^9–11^. Due to its constant exchange with the tumor, the tdLN is additionally exposed to tumor-mediated immune microenvironment remodeling^12,13^. Together, these effects result in a size increase of the tdLN that is observed in both human patients and mouse models of cancer^14–16^.

In the tdLN, neutrophils are emerging as an important player in tumor-immune dynamics^17,18^. Traditionally, neutrophils have been thought of as short-lived effector cells that patrol the blood and rapidly enter damaged or infected tissues^19^. Over the last two decades, their more complex roles in cancer and in shaping the immune environment of lymph nodes have become increasingly apparent. Neutrophils play a dual role in cancer, anti-tumoral in early and pro-tumoral in late stages^17,18,20^. In the tumor micro-environment (TME), tumor-associated neutrophils or polymorphonuclear myeloid-derived suppressor cells are associated with reduced T cell function and worsening treatment outcomes in many solid cancers^21–23^. Neutrophils are known to interact with CD8^+^ T cells in the TME of various cancers and are recognized as modulators of T cell function during ACT^24,25^. These interactions can lead to reduced fitness and an impaired anti-tumor response^26–28^. While present in small numbers during homeostasis^29^, neutrophils infiltrate both draining and non-draining lymph nodes during cancer progression^30^. In the tdLN, neutrophils can acquire an immunosuppressive phenotype similar to their counterparts in the TME, as shown in samples from patients with head and neck cancer (HNC)^17^ and in a mouse model of melanoma treated with ACT^25^. Neutrophils infiltrating draining lymph nodes during infection do not enter the parenchyma^31^. Instead, they localize to the medulla and, when activated, enter the interfollicular zone^31^. In the context of early stage HNC, it was found that neutrophil accumulation in T cell rich zones of metastasis-free tdLNs was positively correlated with five-year survival in HNC patients^17^. However, during disease progression, neutrophils increasingly acquired an immunosuppressive phenotype in response to tumor-derived signals^17^. How these context-dependent findings translate to neutrophil dynamics and spatial organization in tdLN versus non-tdLN during ACT is not yet clear.

Here, we investigate the reactive neutrophil response and the neutrophil–CD8^+^ crosstalk in the lymph nodes during ACT to obtain a more holistic understanding of the cancer-immune interaction. We leverage a murine melanoma model with ACT as previously described^25,32^. We longitudinally quantify neutrophils and adoptively transferred CD8^+^ T cell subset dynamics over a 14 day period after treatment initiation in lymph nodes from three distinct anatomical regions. Our results suggest that neutrophils have an increased relative abundance and a distinct spatial distribution in the non-tdLN as compared to the tdLN. An increased neutrophil abundance was positively associated with terminally differentiated effector CD8^+^ T cells (T_EFF_), as compared to the longer-lived central memory CD8^+^ T cells (T_CM_ cells). Furthermore, we show that innate immune signaling in the tumor promotes neutrophil responses, and importantly augments differences in neutrophil infiltration between the tdLN and non-tdLN.

## Results

### Neutrophils infiltrate predominantly into the distant contralateral lymph node (clLN) compared to the tdLN during ACT

**Fig. 1:**
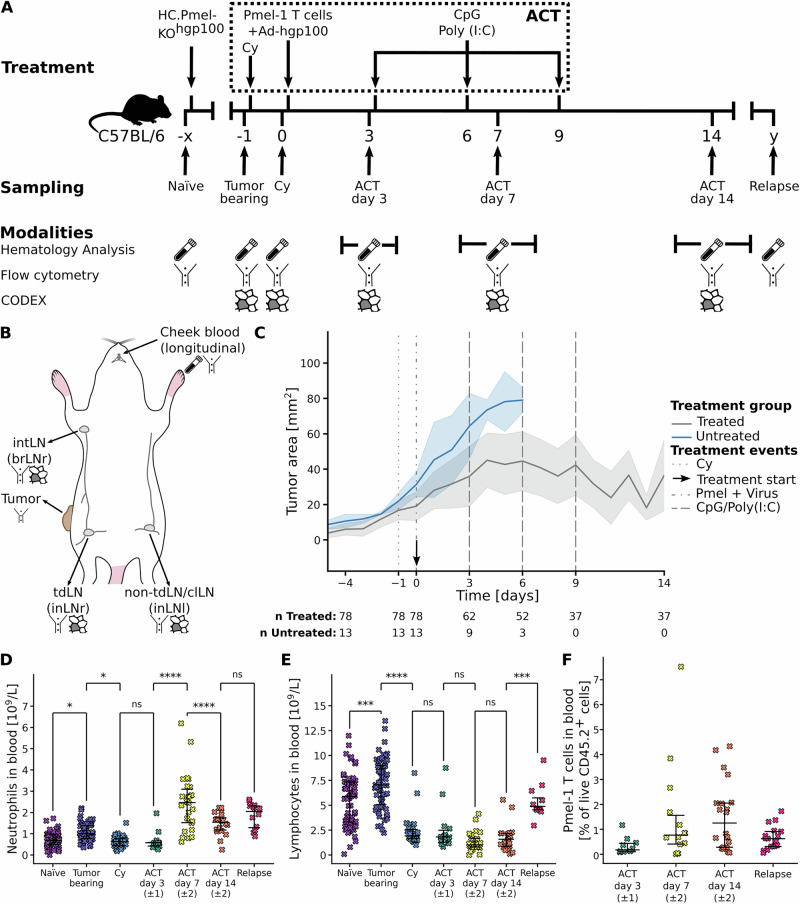
Assessment of tumor establishment and Pmel-1 T-cell engraftment upon ACT. **A** Experimental setup and sampling strategy of Pmel-1 ACT in C57BL/6 mice bearing HC.PmelKO.CDK4R24C-NFhgp100 melanomas. Cy: cyclophosphamide treatment. Ad-hgp100: recombinant adenovirus expressing human gp100. CpG/Poly(I:C): CpG/Polyinosinic:polycytidylic acid, innate immune ligands. The complete ACT treatment is indicated within the dashed box. Time points x and y vary between mice. **B** Schematic of mouse with lymph nodes and tumor indicated. tdLN: tumor-draining lymph node (inLNr), intLN: intermediate lymph node (brLNr), non-tdLN: non-tumor-draining lymph node (clLN or inLNl). Icons indicate the modalities obtained per tissue as in **A**. **C** Tumor growth trajectories of all mice. Mice in the treatment group received ACT at indicated time points and were sacrificed at the sampling time points in **A**. Untreated mice were sacrificed when their tumor reached 8–10 mm in diameter. The shaded area indicates ± 1 standard deviation from the population mean. **D, E** White blood cell quantification in cheek blood for neutrophils **D** and lymphocytes **E** using Mindray BC-5000 Vet Hematology analyzer. **F** Pmel-1 T cell concentrations in the blood at sacrifice, measured using flow cytometry. Unpaired tests in **D-F** performed using ANOVA with Tukey’s honestly significant difference (HSD) for family-wise error correction. ****: *p* < 0.0001, ***: *p* < 0.001, **: *p* < 0.01, *: *p* < 0.05 and ns: p≥0.05. Non-significant associations in **F** are not shown.

To investigate the dynamics of different immune cell populations during ACT, C57BL/6 mice were subcu-taneously (s.c.) inoculated with a syngeneic melanoma cell line (HC.PmelKO.CDK4R24C-NFhgp100) that was previously established by a method termed CRISPitope (CRISPR-assisted insertion of epitopes)^33,34^ (Fig. 1A). CRISPitope enables the generation of tumor cells expressing model CD8^+^ T cell epitopes fused to endogenously encoded gene products of choice. In this case, the endogenously expressed oncogenic allele of CDK4 (CDK4R24C) was rendered immunogenic in a neoepitope-like manner by fusing the human gp100 model epitope (hgp100) to its C-terminus. Pmel, the gene encoding for the mouse gp100 was ablated by CRISPR-Cas9 (HC.PmelKO) prior to CRISPitope engineering. Even though the mouse gp100 epitope poorly binds to H2-Db, the respective MHC class I allele in C57BL/6 mice, we considered the knockout of gp100 (Pmel) as preferable because it established a defined genetic background for our ACT protocol. Monoclones from the polyclonal culture of the HC.PmelKO.CDK4R24C-NFhgp100 melanoma cell line^34^ were then established and used in the present study. ACT was started when tumor size reached 3–5 mm in diameter. The ACT treatment consisted of a single round of cyclophosphamide (Cy) treatment, followed the next day by intravenous (i.v.) injection of 2 million Pmel-1 T cells, recognizing the hgp100 epitope presented by H2-Db and isolated from spleens of naïve Pmel-1 T-cell receptor (TCR) transgenic mice. Pmel-1 T cell injection was accompanied by in vivo activation of the cells by recombinant adenoviral vector Ad-hgp100 as previously described^34–36^. Pmel-1 T cells specifically recognize the hgp100 epitope presented by H2-Db. The treatment was completed with innate immune activation via intratumoral (i.t.) injection of CpG/Polyinosinic:polycytidylic acid (CpG/Poly(I:C)). Cohorts of mice were sampled prior to tumor challenge (Naïve), when tumors reached a diameter of 3-5 mm (Tumor-bearing), one day post-Cy treatment (Cy) and at days 3, 7, and 14 after the start of ACT, as well as upon relapse. Three lymph nodes from distinct anatomical regions were collected from each mouse (Fig. 1B) and each lymph node was used for either multi-parametric flow cytometry or multiplex immunofluorescence (Supplementary Table S1). The lymph nodes considered in this study are: i) the inguinal lymph node left (inLNl), which is the tumor-draining lymph node (tdLN) (tdLN) in this model, ii) the inguinal lymph node left (inLNl), which is the contralateral lymph node (clLN) to the tdLN and thus considered the non-tumor-draining lymph node (non-tdLN), and finally iii) the brachial lymph node right (brLNr), which is the intermediate lymph node (intLN) between the tdLN and the clLN considering its position distant from the tdLN but belonging to the same ipsilateral lymphatic draining basin. Hereafter, will refer to lymph nodes using the functional, drainage-based nomenclature. To determine the time point for the treatment initiation and to monitor mouse well-being, tumor size was measured three times per week.

Our assessment of the tumor size revealed varying dynamics between mice. Around 20% of mice never developed a tumor or had minor tumor growth, but cleared it before it reached the treatment size (Supplementary Figure S1, Supplementary Table S1). In the remaining 80%, established tumors responded to ACT and were effectively controlled until day 14 (Fig. 1C). For the majority of mice, tumor control extended past ACT day 14, with an average time between post-treatment response and relapse of 19 days (Supplementary Table S3). Among the 17 mice with initial tumor development in the Relapse cohort, one mouse achieved tumor eradication, one mouse showed a transient reduction in tumor burden that did not meet the response criterion, and 15 mice relapsed (Supplementary Table S3, Supplementary Figure S1H). Assessment of white blood cell populations upon Cy treatment revealed an immediate depletion of peripheral blood neutrophil and lymphocyte concentrations (Fig. 1D, E). In contrast, the fraction of those cells in the lymph nodes remained mostly stable upon Cy treatment (Supplementary Figures S2A, B). ACT was followed by an increase in neutrophil count at day 7 post initiation (Fig. 1D). Lymphodepletion in the peripheral blood allowed the Pmel-1 T cells to sustainably engraft in the host, as shown by their presence in the blood (Fig. 1F)^37^.

**Fig. 2:**
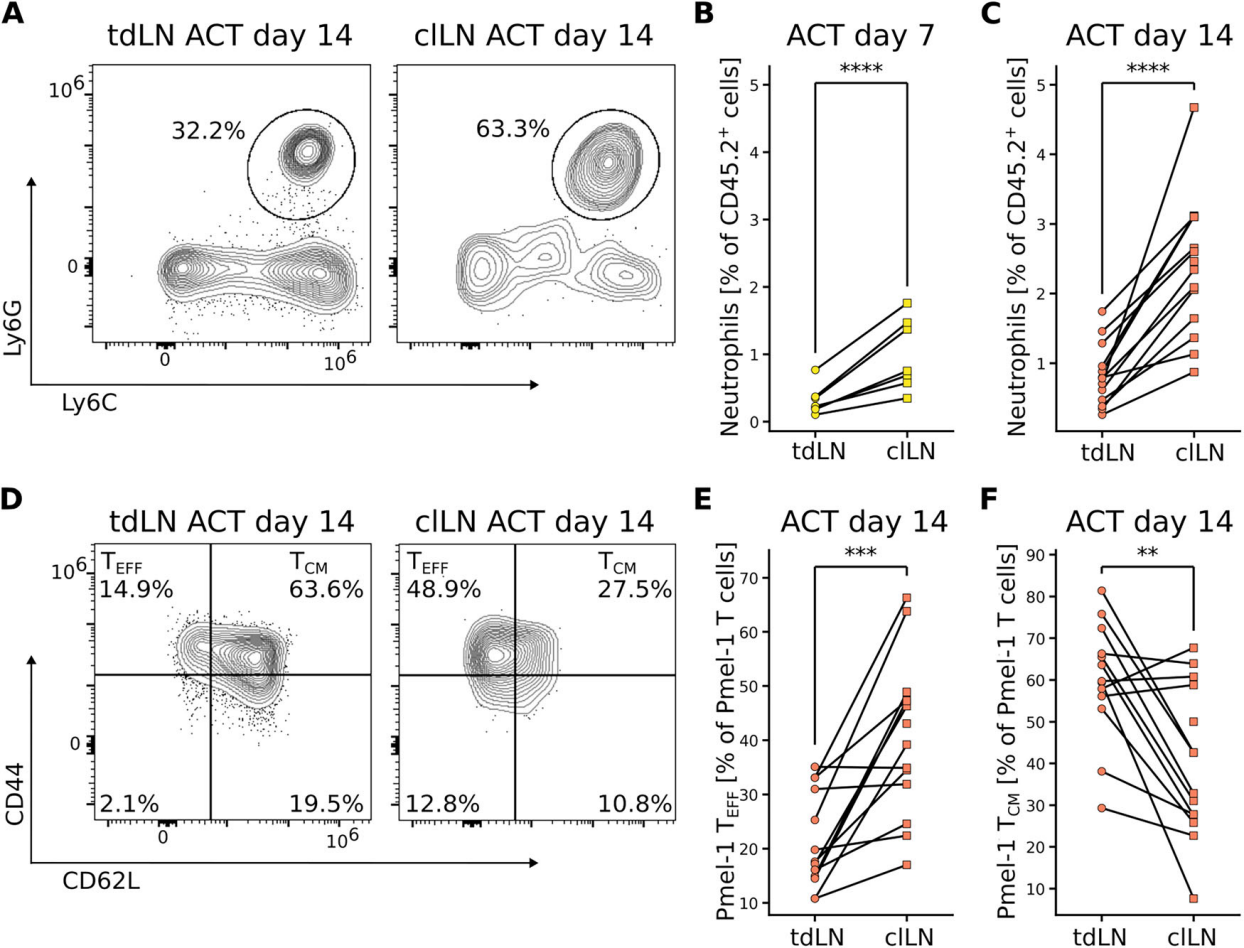
Neutrophil and T cell abundance in the clLN and tdLN. **A** Representative flow cytometric plots showing Ly6C against Ly6G expression on CD45.2^+^CD3^-^ cells for neutrophil phenotyping. **B, C** Quantification of the relative neutrophil abundance in treatment group ACT day 7 **B** and day 14 **C**. **D** Representative flow cytometric plots showing CD62L against CD44 expression on CD45.2^+^CD8^+^CD90.1^+^ cells for CD8^+^ T cell subtyping. **E, F** Quantification of the Pmel-1 T_EFF_ **E** and T_CM_ **F** subsets in treatment group ACT day 14. Paired tests in **B, C, E, F** performed using a ratio-paired T test. ****: *p* < 0.0001, ***: *p* < 0.001, **: *p* < 0.01.

Next, we assessed changes in the lymph nodes and tumor tissue upon ACT. The Pmel-1 T cells accumulated in the lymph nodes between day 3 and 7 of ACT, preferentially the tdLN (Supplementary Figure S2C). The neutrophils also infiltrated the lymph nodes starting from ACT day 3 but mainly between day 7 and 14 (Supplementary Figure S2A). Interestingly, a higher fraction of neutrophils infiltrated the clLN (non-tdLN) compared to the tdLN (Fig. 2A-C). This observation is based on relative cell frequencies and does not necessarily reflect absolute cell numbers, which are analyzed separately below. The intermediate lymph node (intLN) showed a similar but slightly less pronounced trend to the clLN (Supplementary Figure S2A-C), and will be mostly omitted for brevity. The Pmel-1 T cells in the clLN exhibited predominantly T_EFF_ effector phenotypes (CD44^+^CD62L^-^), whereas in the tdLN the central memory T_CM_ phenotype (CD44^+^CD62L^+^) was predominant (Fig. 2E, F)^38^. The T_EFF_ population emerged in the tumor mainly at ACT day 7 and remained the predominant Pmel-1 population there throughout the experiment (Supplementary Figure S2D). In contrast, only approximately 5% of the Pmel-1 T cells in the tumor were T_CM_ cells, increasing in abundance slightly between ACT day 7 and 14 (Supplementary Figure S2E). The T_CM_ phenotype is linked to long-term persistence of transferred T cells^39^. Its predominance among the Pmel-1 phenotypes in the tdLN in our experiments is consistent with this concept.

In summary, we observed neutrophils infiltrating both the tdLN and clLN, whereby their fraction was higher in the non-tdLN (clLN). The tdLN contained a higher fraction of beneficial T_CM_ cells.

### Spatial analysis reveals enhanced T cell zone infiltration in the tdLN compared to the clLN

**Fig. 3:**
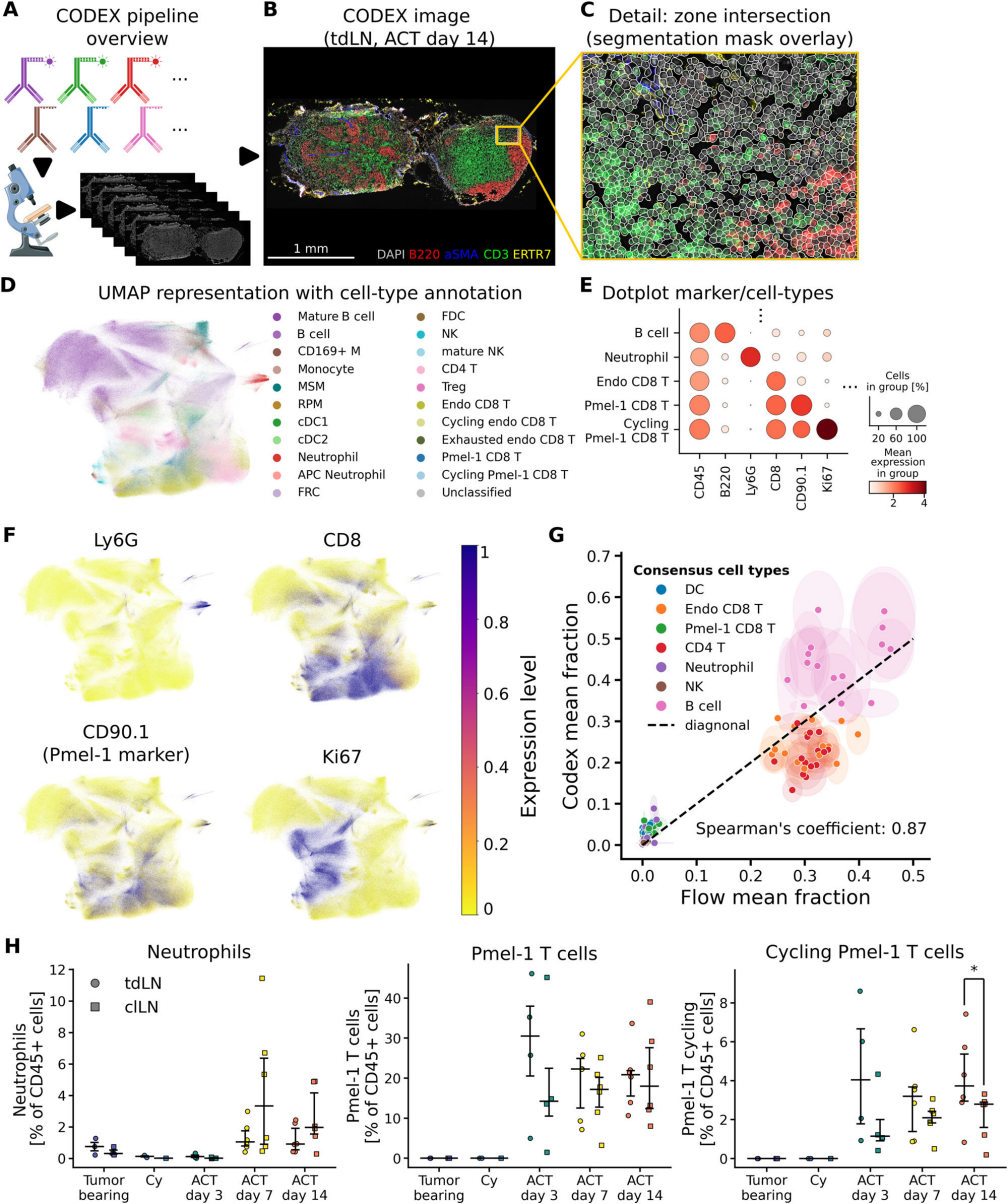
Multiplex immunofluorescence image analysis of lymph nodes. **A-E** Overview of the experimental **A** and computational **B-E** pipeline. **B** tdLN from ACT day 14. Selected markers: DAPI (grey), B220 (red), aSMA (blue), CD3 (green), ERTR7 (yellow). **C** Segmentation mask over detail at zone intersection of T cell zone, B cell zone and Medulla of the tdLN in **B**. **D, E** Sample full UMAP embedding **D** and dotplot **E** of all multiplex immunofluorescence imaging samples, showing cell type annotation. Dot color in **E** is the normalized mean marker expression in the cell type and dot size indicates the fraction of cells expressing the marker in that cell type. **F** Expression level of key markers embedded in UMAP space. **G** Cross-modality expression alignment. Each dot represents a condition-lymph node combination. **H** Quantification of neutrophil and Pmel-1 T cells in the tdLN and clLN per experimental condition in the imaging data. Paired tests in **H** performed using a ratio-paired T test. *: *p* < 0.05. Non-significant associations are not shown. Icons in **A** are adapted from Servier, licensed under CC BY 3.0.

To elucidate the role that the neutrophils play in the lymph nodes upon ACT and to find out how they interact with CD8^+^ T cells, we investigated them in their spatial context during the course of ACT treatment. To this end, we performed multiplex immunofluorescence imaging using the PhenoCycler (formerly co-detection by imaging (CODEX)) platform on the remaining lymph nodes by applying a broad immune cell panel of thirty markers for state and functional phenotyping (Fig. 3A-F, Supplementary Figure S3A).

This analysis identified 22 cell types in their spatial context (Fig. 3D), with a clear Ki67^+^ CD90.1^+^ (Pmel-1) compartment defining cycling Pmel-1 CD8^+^ T cells (Fig. 3E, F). Comparison of immune cell populations between the imaging and flow cytometry data revealed a good general alignment, with a Spearman’s rank correlation coefficient of 0.87 (Fig. 3G). We found that B cells were slightly overrepresented in the imaging data and T cells were slightly underrepresented as compared to the quantification by flow cytometry. In the organ-level distribution of the imaging cell types, the neutrophils and Pmel-1 T cells showed similar trends to their counterparts in the flow cytometry data: Neutrophils appeared more abundant in the clLN and cycling Pmel-1 T cells more in the tdLN (Fig. 3H).

**Fig. 4:**
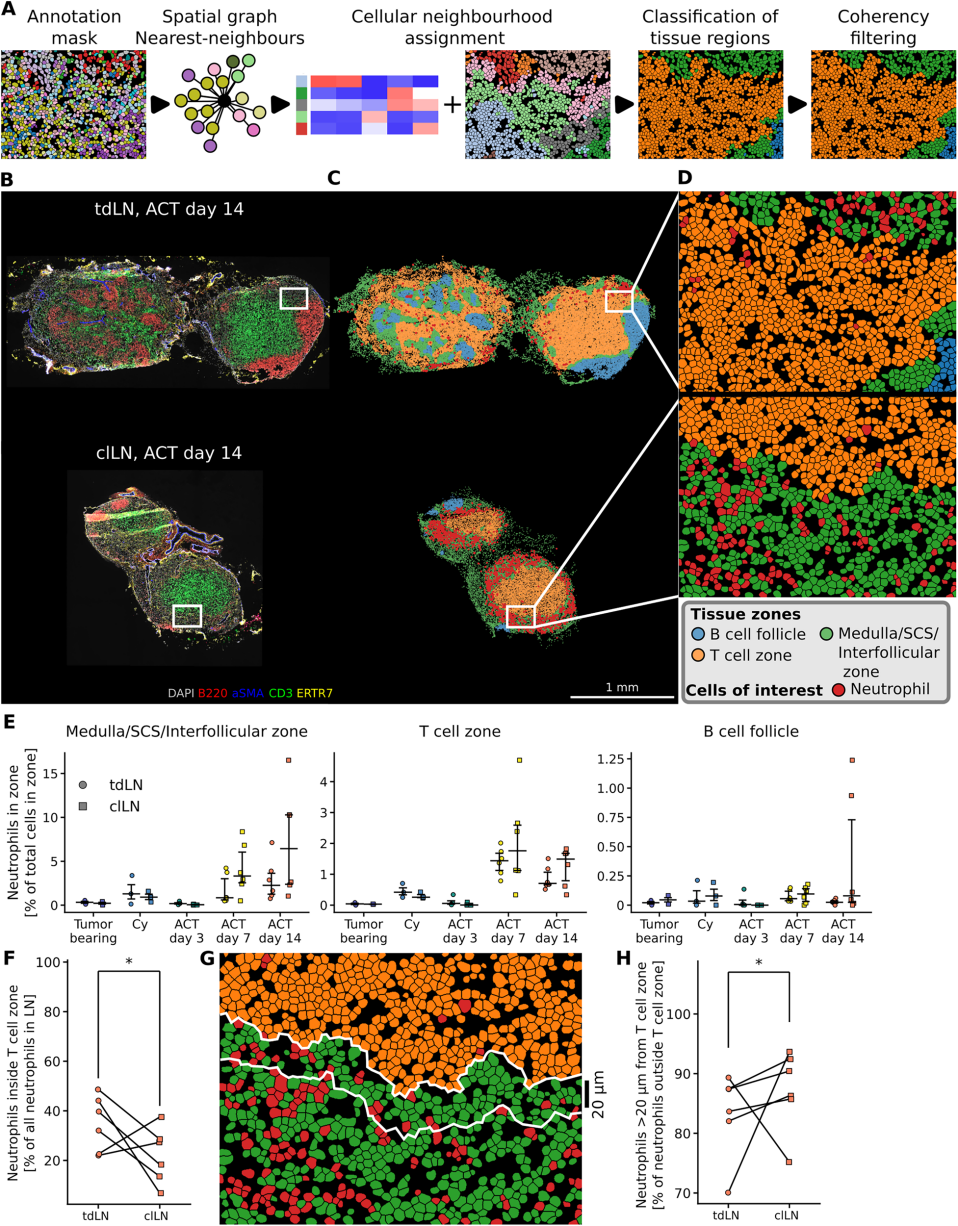
Analysis of neutrophil localization in lymph nodes. **A** Overview of spatial analysis workflow. **B** Representative tdLN and clLN at ACT day 14, to scale. Selected markers: DAPI (grey), B220 (red), aSMA (blue), CD3 (green), ERTR7 (yellow). **C** Zone classification of the two lymph nodes in **B**. Neutrophils are indicated in red. **D** Zoom of **C** with region annotation and neutrophils. **E** Neutrophil population in the three tissue zones as a fraction of total cells per zone. **F** Neutrophils present in the T cell zone as percentage of all neutrophils, ACT day 14. **G** Zoom of clLN from **D** with T cell border and 20 *μ*m distance from border indicated in white. **H** Neutrophils outside the T cell zone at a 20 *μ*m distance from the border, as a percentage of neutrophils outside the T cell zone. Unpaired tests in **E** performed using ANOVA with Tukey’s HSD for family-wise error correction. Non-significant associations in **E** are not shown. Paired test for non-normally distributed data in **F** performed using Wilcoxon signed-rank test. Paired test for normally distributed data in **H** using a ratio-paired T test with Holm correction across tested distance thresholds. *: *p* < 0.05.

Beyond the relationship between lymph nodes from distinct anatomical regions, it is important to investigate the spatial distribution of neutrophils within them. To this end, the lymph nodes were classified into three regions based on a spatial graph and cellular neighborhood assignment: the T cell zone, B cell follicle, and an intermediate zone comprised of the medulla, subcapsular sinus (SCS) and the interfollicular zone. These three regions were subsequently smoothed by iterative majority voting among neighboring cells until effective convergence (Fig. 4A, Supplementary Figures S4, S5). Comparing a representative tdLN and clLN at day 14 (Fig. 4B), we observed marked size differences between them. Additionally, in line with the flow cytometry analyses, we observed a higher fraction of neutrophils in the clLN, which appear to be localized mainly in a wide ring outside the T cell zone (Fig. 4C, D). Exploring this observation further, our quantification across the treatment groups revealed that neutrophil presence was most enhanced in the medulla/SCS/interfollicular zone (intermediate zone) (Fig. 4E). In this zone, they comprised six percent of the total cells in the clLN and three percent in the tdLN on average at day 14 after treatment start.

In some mice of the late-stage ACT groups, neutrophils accounted for almost 10% of the total cells in this intermediate zone in the clLN (Fig. 4E). They also entered the T cell zone, though here they constituted a smaller fraction of this zone. They were hardly present in the B cell follicle. When comparing the lymph node localization at day 14 of ACT, a higher fraction of neutrophils was detected in the T cell zone in the tdLN compared to the clLN (Fig. 4F). Among the neutrophils that were outside of the T cell zone, those in the clLN seemed to distance themselves from the T cell zone, appearing predominantly more than 20 *μ*m (approximately five cell diameters) away from the T cell zone border (Fig. 4G, H).

In summary, neutrophils localized mainly outside the T cell zone but were more commonly found there in the tdLN than the clLN. In the clLN, they instead maintained a distance of approximately five cell diameters from the T cell zone.

### Profound changes in lymph node sizes and architecture facilitate immune response in ACT

The tdLN at day 14 of ACT was enlarged to about two times the diameter of the clLN at that same time point (Fig. 4B). Establishing how lymph node size and growth dynamics correspond to the immune response is crucial for interpreting the response’s functional impact on the organism. To this end, we mapped the growth of the lymph nodes and their anatomical zones over time and between lymph nodes.

**Fig. 5:**
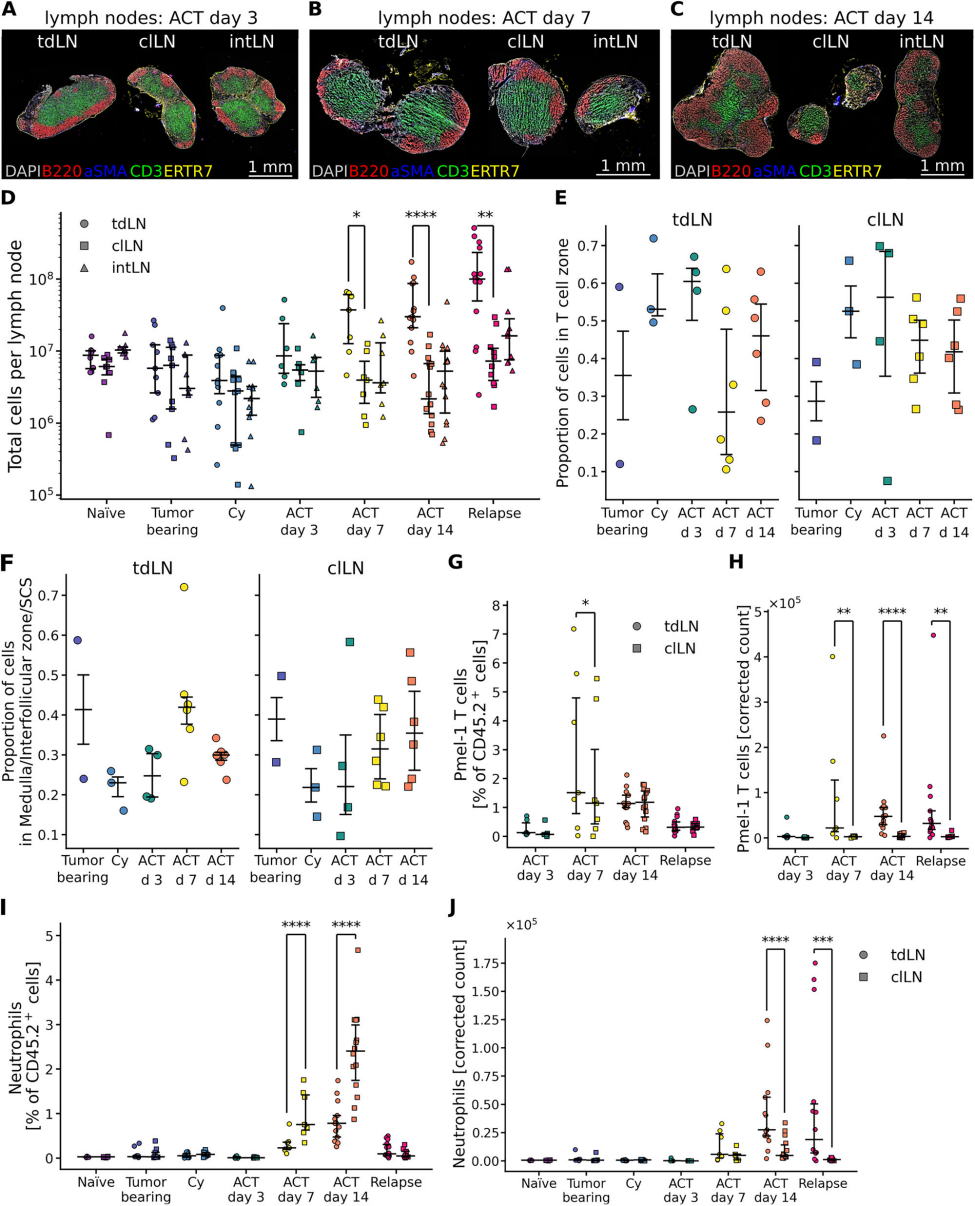
Lymph node size and tissue architecture differences. **A, B, C** Lymph node multiplex immunofluorescence imaging images from three representative mice at ACT day 3, 7 and 14. Selected markers: DAPI (grey), B220 (red), aSMA (blue), CD3 (green), ERTR7 (yellow). **D** Quantification of lymph node size differences based on total live cell counts in flow cytometry. **E, F** Proportion of total cells present in the T cell **E** and intermediate **F** zones, as measured by multiplex immunofluorescence imaging. **G-J** Pmel-1 T cells **G, H** and neutrophils **I, J** as a fraction of CD45.2^+^ cells **G, I** and as bead-corrected counts **H, J** in the tdLN and clLN, measured using flow cytometry. Paired tests between tdLN and clLN **D, G, H, I, J** performed using a ratio-paired T test. Unpaired tests **E, F** performed using ANOVA with Tukey’s HSD for family-wise error correction. ****: *p* < 0.0001, ***: *p* < 0.001, **: *p* < 0.01 and *: *p* < 0.05. Non-significant associations are not shown.

Our assessment of bead-corrected live cell counts in flow cytometry revealed that Cy conditioning was associated with a modest, statistically non-significant reduction in lymph node size across all nodes (Supplementary Figure S6A). Despite the size decrease, the lymph node architecture did not change visibly under Cy conditioning (Supplementary Figure S6B). As Cy is known to preferentially kill proliferating cells^40,41^, we assessed Ki67 expression in multiplex immunofluorescence images before and after Cy administration (Supplementary Figure S7A). We observed a non-significant trend towards reduced Ki67 expression following Cy treatment, with no evidence for differential effects across lymph nodes within a condition (Supplementary Figure S7B, C). At later time points the tdLN grew under the effect of ACT and continued tumor presence (Fig. 5A-C). From the naïve to the relapse time points, the tdLN grew approximately by one order of magnitude from 10^7^ to 10^8^ cells on average (Supplementary Figure S6A). The clLN and intLN sizes remained stable during the course of the experiment. The exception to this was the intLN growing significantly between the ACT day 14 and relapse time points. The tdLN contained significantly more cells as compared to the other two lymph nodes from ACT day 7 onward (Figure 5D). At relapse, the tdLN contained an order of magnitude more cells than the clLN.

We examined which lymph node anatomical zones contributed to the observed increase in cell number. The proportion of cells that make up the T cell zone increased early after treatment start. At ACT days 7 and 14, this growth was overshadowed by the intermediate zone in both the tdLN and clLN (Fig. 5E, F). However, variability between mice was large and no significant correlations could be observed. The B cell follicle remained proportionally stable throughout treatment (Supplementary Figure S6C, D). These size differences mean that what seems to be a small effect when shown as a percentage could actually obscure a large increase or decrease in an immune cell population, with possible profound biological implications. For example, the fraction of Pmel-1 T cells within the CD45.2^+^ population was similar between the tdLN and the clLN at day 14 and at relapse (Fig. 5G). In contrast, bead-corrected absolute counts revealed that Pmel-1 T cells were significantly more abundant in the tdLN at ACT days 7 and 14 and at relapse (Fig. 5H). This underscores the importance of the tdLN as main reservoir of the anti-tumor T cell population. The neutrophils showed an opposite trend: while they were relatively more abundant in the clLN at day 7 and 14 (Fig. 5I), their absolute counts remained lower than those observed in the rapidly growing tdLN (Fig. 5J). In the relapse condition, the neutrophil fraction was comparable between the lymph nodes.

To summarize, we observed significant size increases of the tdLN during ACT and emphasized the need to take absolute cell abundances into account when assessing the effect of expanding immune cell populations on the organism.

### Intratumoral innate immune stimulation triggers infiltration of neutrophils in the lymph nodes and promotes T_CM_ cells in the tdLN

**Fig. 6:**
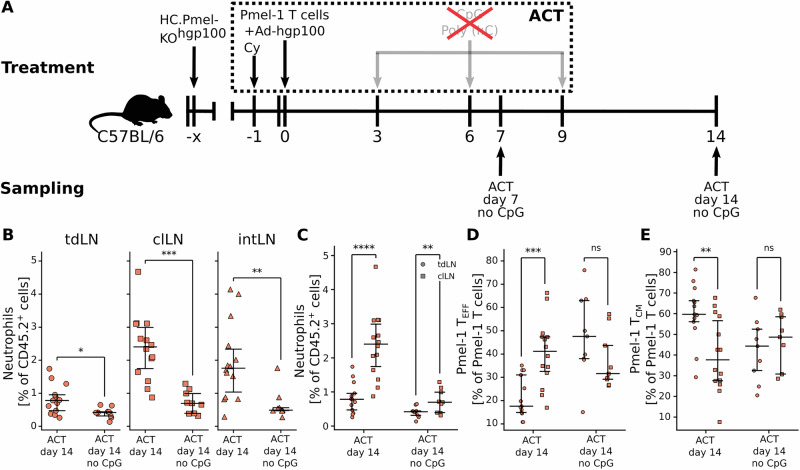
ACT without innate immune stimulation (sampling at ACT day 7 and 14). **A** Experimental setup and sampling strategy of Pmel-1 ACT without CpG/Polyinosinic:polycytidylic acid (CpG/Poly(I:C)) treatment in C57BL/6 mice bearing HC.PmelKO.CDK4R24C-NFhgp100 melanomas. **B** Neutrophil abundance with and without CpG/Polyinosinic:polycytidylic acid (CpG/Poly(I:C)) treatment at day 14, per lymph node. **C–E** Neutrophil, T_EFF_ and T_CM_ concentrations in the lymph nodes at day 14 of ACT with and without innate immune stimulation. Unpaired test in (**B**) performed using ANOVA with Tukey’s HSD for family-wise error correction. Paired tests (**C-E**) performed using a ratio-paired T test. ****: *p* < 0.0001, ***: *p* < 0.001, **: *p* < 0.01, *: *p* < 0.05 and ns: p≥0.05. All data was obtained using flow cytometry.

We observed previously that fewer Pmel-1 T cells in the clLN exhibited a beneficial T_CM_ phenotype and that the clLN had a higher neutrophil fraction (Fig. 2). It remained unclear whether this shift was caused by a difference in tumor antigen exposure between these lymph nodes, by a different level of exposure to i.t. CpG/Polyinosinic:polycytidylic acid (CpG/Poly(I:C)) innate immune injection, or both. To investigate this, we omitted the CpG/Polyinosinic:polycytidylic acid (CpG/Poly(I:C)) stimulation at day 3, 6 and 9 of the ACT protocol and sampled tissues at ACT day 7 and 14 (Fig. 6A). Importantly, previous work established that i.t. CpG/Polyinosinic:polycytidylic acid (CpG/Poly(I:C)) application is in particular critical for the long-term immune surveillance capacity of this combinatorial ACT regimen^35^.

Our assessment of tumor burden in the no-CpG/Polyinosinic:polycytidylic acid (CpG/Poly(I:C)) condition revealed similar control of tumor growth as compared conditions with CpG/Polyinosinic:polycytidylic acid (CpG/Poly(I:C)) until day 14 (Supplementary Figures S1J-L). This is not in disagreement to published work, because the short time window precludes conclusions about long-term therapeutic effects. Lymph node sizes were also similar with and without innate immune stimulation (Supplementary Figure S8A). Omission of innate immune stimulation reduced the neutrophil abundance in all lymph nodes (Fig. 6B, in comparison with Fig. 5D). Additionally, the pronounced difference in the neutrophil fraction between the tdLN and the clLN was reduced at ACT day 14 without CpG/Polyinosinic:polycytidylic acid (CpG/Poly(I:C)) (Fig. 6C, Supplementary Figure S8B). Among the Pmel-1 T cells, the T_EFF_ showed a similar trend. When omitting innate immune stimulation, at day 14 the difference in T_EFF_ abundance between the tdLN and clLN lymph nodes disappeared (Fig. 6D). This was mainly due to the significant increase of the T_EFF_ fraction in the tdLN under this condition (Supplementary Figure S8C). In addition, the differences in T_CM_ concentration proportions between the clLN and tdLN also disappeared at ACT day 14 without innate immune stimulation (Fig. 6E, Supplementary Figure S8D). Interestingly, despite these changes in the lymph nodes, the T_EFF_ and T_CM_ populations in the tumor were unchanged (Supplementary Figures S8E, F).

To summarize, omitting innate immune stimulation did not change tumor growth dynamics early after treatment start until day 14. It also did not change Pmel-1 T cell populations in the tumor or lymph node sizes. However, without innate immune stimulation, the reactive neutrophil infiltration into all lymph nodes was reduced, which was accompanied by an increase of the T_EFF_ population and a decrease of the T_CM_ population in the tdLN. Thus, our results revealed that i.t. innate immune activation by CpG/Polyinosinic:polycytidylic acid (CpG/Poly(I:C)) is important to promote the establishment of the favorable Pmel-1 T_CM_ population in the tdLN, which is known to be critical for long-term persistence.

## Discussion

In the tdLN under ACT, neutrophils acquire an immunosuppressive phenotype^25^. However, a holistic understanding of neutrophil behavior during ACT and the neutrophil-CD8^+^ T cell crosstalk is still incomplete. To address this, we conducted a multi-compartment investigation of lymph nodes from distinct anatomical regions, quantifying neutrophil and Pmel-1 T cells in three different lymph nodes in melanoma-challenged mice treated with ACT. In particular, we focused on the early on-treatment window until day 14 after ACT start to capture the immune cell dynamics in the expansion phase of the adoptively transferred Pmel-1 T cells.

Among the three lymph nodes, we observed an enhanced neutrophil response in the non-tdLN (clLN). This was contrary to our expectation, because we assumed that the tumor-derived cytokine milieu promotes neutrophil infiltration into the tdLN. Previous studies, including ours, have found that neutrophils in tumor-draining lymph nodes can acquire immunosuppressive properties^24,25,30,42,43^, but deeper neutrophil phenotyping was not included in our current study. Moreover, whether neutrophil recruitment to the non-tdLN influences tumor immune surveillance remains speculative. Addressing this question would require tailored experimental setups to dissect the respective functional contributions of the tdLN and non-tdLN, for which our study was not designed. In other contexts, immunosuppressive neutrophils can influence functional T cell differentiation either indirectly by promoting CD4^+^ cells to differentiate into effector regulatory T cells^44,45^ or directly, by contact-dependent suppression of CD8^+^ T cell functions^17,25,46,47^. Accordingly, this leads to reduced fitness of the T cell population and an impaired anti-tumor response^21,24,26–28^.

Following our initial observation of enhanced neutrophil presence in the clLN, we investigated the spatial distribution of the neutrophils within the lymph nodes. We found that the majority of neutrophils were localized outside of the T cell zone, mainly in the medulla. In the tdLN, neutrophils were more likely to be in the T cell zone than their counterparts in the clLN, which also kept a larger relative distance from the T cell zone. Based on their spatial distribution, our data suggest that the neutrophils in the tdLN may have more interactions with T cells. These neutrophils could enable immunosuppressive functions via PD-L1 and other mechanisms, as shown in our previous work^34^. However, this interpretation is based on spatial proximity and cannot be directly assessed with the existing dataset. Indeed, neutrophils in the T cell zones of the tdLN may have both pro - and anti-tumoral roles depending on their phenotype and stage of the disease, as was previously observed in tdLN samples from HNC patients^17^. The presence of HLA-DR^+^CD80^+^CD86^+^ICAM1^+^PD^-^L1^-^ neutrophils in the T cell zone was associated with favorable disease outcome, whereas PD-L1^+^ neutrophils predicted poor outcome^17^. Another study found distinct neutrophil subsets in human draining and non-draining lymph nodes for patients with oral squamous cell carcinoma^30^. Here, non-draining lymph nodes contained CD16^high^CD62^high^ neutrophils (mature and non-active), whereas tdLN contained more CD16^high^CD62^dim^ (mature and hyper-activated) neutrophils^48^, whose abundance correlated with tumor stage^30^. Given the high plasticity of neutrophils, such dual and context-dependent effects are not surprising.

Using the total amount of live cells measured in flow cytometry as a proxy, we calculated the growth of the tdLN during the treatment time course and compared it to the other lymph nodes. We found that the tdLN rapidly increased in size, up to an order of magnitude larger than its counterparts at the relapse time point. Taking LN size and total cell numbers into account is important to interpret cell frequencies properly, as shown for the adoptively transferred Pmel-1 T cells. Their total number was much higher in the tdLN, whereas their frequency remained comparable between the tdLN and non-tdLN.

Lymph node architecture also appeared to be altered, with relatively slower growth of the T cell zone compared to the intermediate zone. An important caveat to this finding is that compositional changes were estimated from 2D tissue slices. While these were cut in the same way each time to be as representative as possible, 2D imaging remains a tenuous modality for this purpose^49,50^. An example of the uncertainty this introduced becomes apparent when comparing the live cell counts from flow cytometry and cell counts per zone in multiplex immunofluorescence data. In flow cytometry, the total amount of live cells appeared to decrease upon Cy treatment. This was followed by a steady growth, though the size increase was not significant between conditions up to the relapse time point, a kinetic that is very plausible following chemotherapeutic conditioning. By contrast, the cell counts estimated from multiplex immunofluorescence indicated that lymph nodes increased in size upon Cy treatment. However, the sample size was much lower than for flow cytometry and each section may be subject to sample bias. Because we were aware of this weakness in our multiplex immunofluorescence data, we focused on interpreting relative, compositional changes. We only loosely interpreted absolute differences in this modality, as immune cell frequencies were quite robust between flow cytometry and multiplex immunofluorescence.

Next, we investigated the neutrophil response when omitting the intratumoral innate immune stimulation in the tumor and found that the neutrophil response was diminished in the clLN as compared to the tdLN. However, the relative abundance difference did not disappear entirely. This suggests that the strong neutrophil response in the clLN was caused to a large extent by the innate immune stimulation. One possible explanation is that the tdLN, due to enhanced exposure to tumor-derived signals, is functionally conditioned in a way that alters its responsiveness to acute inflammatory cues, for example through changes in its chemokine milieu or vascular activation state. Though the precise mechanism remains unclear, another potential explanation is that intratumoral CpG/Polyinosinic:polycytidylic acid (CpG/Poly(I:C)) treatment leads to a stronger increase of type I interferon levels in the tdLN, where type I interferon may suppress CXCL2 production, a major chemokine driving neutrophil recruitment via CXCR2^51,52^. Additionally, differences in lymphatic drainage patterns between lymph nodes may further contribute to the observed asymmetry.

The T_CM_ population in the tdLN, previously associated with long-term engraftment^8^, was decreased when omitting innate stimulation of the melanoma. This concurrent reduction in neutrophil abundance and T_CM_ frequency raises the question of whether neutrophils directly contribute to T cell differentiation, or whether both effects reflect parallel consequences of diminished innate immune activation. While neutrophils have been reported to modulate T cell responses in certain contexts^17,25,46,47^, our data do not allow us to infer a direct causal relationship. An alternative hypothesis is that CpG/Polyinosinic:polycytidylic acid (CpG/Poly(I:C))-induced innate signaling directly supports T_CM_ differentiation, since it has many favorable effects on antigen processing and presentation that are critical for promoting local and systemic T cell responses. Further work will be required to disentangle these potential mechanisms. Importantly, this observation does not alter the main conclusions of our study, but rather highlights that innate immune stimulation simultaneously shapes both neutrophil and T cell compartments.

These findings may have implications for clinical ACT, where lymph nodes are key sites of T cell priming and maintenance. Our data suggest that such treatments may differentially shape immune composition across lymph nodes. However, differences between murine models and patients limit direct translation, and further studies are required to assess whether similar dynamics occur in the clinical setting. Nevertheless, our work highlights the importance of systemic immunity and aligns with recent clinical advances achieved with neoadjuvant immune checkpoint inhibition across multiple cancer types, further underscoring the critical role of tdLNs in reinvigorating anti-tumor immune responses^53,54^.

In summary, we showed that adoptively transferred tumor-reactive CD8^+^ T cells rapidly expanded after ACT onset, with the highest proportion of a favorable T_CM_ phenotype in the tdLN. Augmenting innate immune signaling in the tumor promoted neutrophil recruitment, most prominently in non-tdLN. However, the presence of neutrophils in the T cell zone was more pronounced in the tdLN, which also harbored the largest absolute pool of transferred CD8^+^ T cells. Collectively, these data reveal distinct early neutrophil kinetics and spatial organization in tdLN versus non-tdLN during ACT, influenced by innate immune signaling in the tumor. Our work, focusing on the lymph node compartment during ACT, underscores the dynamic behavior, complexity and context-dependency of neutrophil responses. We share all relevant resources to facilitate subsequent analysis as well as modeling studies. This study highlights the importance of expanding beyond the conventional tumor-tdLN axis towards considering the lymphatic system as a whole.

## Methods

### Cell lines

The parental HCmel12 melanoma cell line was established from a primary tumor in the HGF-CDK4^R24C^ mouse as previously described^36^. To generate the HC.PmelKO melanoma monoclonal cell line, the Pmel gene was stably knocked-out in the HCmel12 cell line by CRISPR-Cas9 and subsequently re-established as an ex vivo cell line to ensure a more homogenous growth in vivo^34^. As previously described, the ex vivo HC.PmelKO cell line was then modified using the CRISPitope approach to insert CDK4(R24C) protein containing Neon(N) - Flag(F) tag - hgp100 - T2A - Puromycin resistance gene. Monoclones from these cells (B2) and an ex vivo derivative of B2 (1923) were established. The B2 or 1923 cell lines were used in these experiments.

All melanoma cell lines were cultured in Roswell Park Memorial Institute 1640 medium containing GlutaMax (Gibco), further supplemented with 10% Fetal Bovine Serum (FBS) (Gibco), 1% non-essential amino acids (Gibco), 1 mM sodium pyruvate (Gibco), 10 mM HEPES (Roth), 55 *μ*M 2-mercaptoethanol (Gibco), 100 IU/mL penicillin G and 100 *μ*g/mL streptomycin sulfate (Gibco) in a humidified incubator at 37^∘^ C with 5% CO_2_. The cell lines used were routinely tested for mycoplasma contamination by PCR.

### Animals

Wild-type C57BL/6J (H-2^b^) mice used in this study were purchased from Charles River. TCR-transgenic Pmel-1 (B6.Cg-Thy1a/Cy Tg(TcraTcrb)8Rest/J) mice expressing an *αβ* TCR specific for amino acids 25-33 of human and mouse gp100 presented by H2-D^b^ were bred in-house as previously described^25,32,34^.

Mice were housed in individually ventilated cages and humidity-controlled under specific pathogen-free conditions in the animal care facility at the University Hospital Bonn. After one week of in-house acclimatization, experiments were performed with 6–13 week old mice. At the beginning of the experiments, mice were randomly allocated to different experimental cohorts, with attempts to match age, sex and tumor size. However, full sex balancing was not feasible across all groups due to experimental constraints (Supplementary Table S1). Sample size per cohort was determined to ensure sufficient statistical power for both modalities while minimizing animal use and maintaining experimental feasibility. Mice were euthanized by CO_2_ asphyxiation using a gradual fill rate of 20% chamber volume per minute. Animals were continuously monitored during exposure until loss of consciousness and respiratory arrest. CO_2_ exposure was maintained for at least one additional minute after cessation of breathing, and death was subsequently confirmed in accordance with EU Directive 2010/63EG (Annex IV). Since the mice lost consciousness due to CO_2_ exposure prior to cessation of breathing, no separate anesthetic was administered. This method was selected to enable subsequent blood collection by cardiac puncture, which is not feasible following cervical dislocation. All animal experiments were approved by the local government authorities (Landesamt für Verbraucherschutz und Ernährung (LAVE), North Rhine-Westphalia, Germany).

### Subcutaneous tumor inoculation

For tumor inoculation, 2 x 10^5^ HC.Pmelko-NF-hgp100.B2 or HC.Pmelko-NF-hgp100.B2.1923 cells in 100 *μ*L phosphate-buffered saline (PBS) were s.c. injected into the shaved flanks of C57BL/6J mice. Tumor sizes were measured with a caliper three times weekly. Tumor area was calculated with the following equation: area = width x length [mm^2^]. Mice whose transplanted tumors never reached the minimum treatment size of 9 mm^2^ were excluded from further analysis.

### Adoptive T cell therapy

ACT was performed as previously described^34–36^. Briefly, when transplanted tumors reached a mean of 3-5 mm in diameter, mice were injected intraperitoneally (i.p.) with 100 *μ*g of Cy in PBS per gram of mouse. One day post-Cy injection, 2 x 10^6^ naïve Pmel-1 CD8^+^ T cells (in whole splenocytes isolated from Pmel-1 mice) in 200 *μ*L PBS and 5 x 10^8^ plaque-forming units of recombinant adenovirus expressing human gp100.^35,55^ in 100 *μ*l PBS were injected i.v. and i.p., respectively.

At days 3, 6 and 9 post ACT, 50 *μ*g CpG 1826 (MWG biotech) and 50 *μ*g Polyinosinic:polycytidylic acid (Poly(I:C)) (Invivogen) diluted in 100 *μ*L PBS were administered intratumorally.

Mice were euthanized prior to tumor challenge (Naïve), when the tumor reached 3-5 mm or 8-10 mm in diameter (Tumor-bearing or Untreated), one day post-Cy treatment (Cy) and at days 3, 7 or 14 after start of ACT, as well as upon relapse. Additionally, mice were also euthanized when tumors reached 100 mm^2^ or upon signs of illness in adherence with the local ethical regulations.

#### ACT efficacy

ACT efficacy was assessed by measuring tumor growth trajectories on mouse skin. Tumor development, treatment response, tumor eradication and relapse were calculated as follows: Mice who had reached a minimum tumor size of 9 mm^2^ were considered for treatment and subsequent analysis. Tumor eradication was then defined as a tumor burden that, after reaching minimum treatment size, was reduced to 0 mm^2^ for at least two consecutive measurements. The maximum tumor size ${T}_{\max }$ was defined as the largest size measured up to and including ACT day 14. A mouse was classified as responsive to treatment if a tumor size ${T}_{response}\le {T}_{\max }\cdot 0.8$ was measured after the day of ${T}_{\max }$, indicating a 20% reduction in tumor burden. By definition, mice that achieved eradication were also considered responsive. The minimum tumor size ${T}_{\min }$ was measured as the smallest measurement after the day of ${T}_{response}$. Finally, among the mice with either tumor eradication or reduction, the relapse possibility was determined: ${T}_{relapse}\ge {T}_{\min }\cdot 1.2$ and ${T}_{relapse}\ge 9$ for at least two consecutive measurements after the day of ${T}_{\min }$. In addition, tumor size was required to increase monotonically after the relapse event.

### Hematological analysis

Blood samples were collected from the submandibular vein of mice maximum twice weekly up until and at endpoint into Lithium-Heparin-coated Microvette 300 *μ*l tubes (Microvette CB300 LH #16.443) and analyzed on Mindray BC-5000 Vet Hematalogy analyzer. The remaining blood sample in the tubes were then used for flow cytometry analysis. Blood samples were obtained at all treatment time points. For ACT day 3 blood samples were taken within a range of ± 1 days of the time point, for day 7 and 14 within a range of ± 2 days.

### Flow cytometry analysis

For flow cytometry analysis, the lymph nodes and tumors were dissociated mechanically with 5 mL syringe plungers and digested with 1 mg/mL Collagenase D, 20 *μ*g/mL DNase I and 5% FBS in PBS for 30 mins at 37 ^∘^C. The digested cell suspensions were then filtered through 70 *μ*M strainers and washed with FACS buffer (PBS containing 2% FBS). After centrifugation, cells were resuspended in PBS in preparation for staining in 96-well round bottom microplates (TPP, #92697).

Immunofluorescent staining of single cell suspensions was performed according to standard protocols. To block non-specific binding of immunoglobulin to the Fc receptors and to exclude dead cells, cells were first incubated with purified anti-CD16/CD32 antibody (Biolegend, #101302) and Live/Dead Blue fixable dye (Thermo Fisher #L23105) for 15 mins at room temperature in the dark. After washing once in FACS buffer, the cells were stained with the following fluorophore-conjugated monoclonal antibodies specific for mouse: CD45.2, CD4, Ly6G, B220, CD62L, CD8a, CD44, CD11c, CD3, CD90.1, XCR1, MHCII, CD11b, NK1.1, CD25 and Ly6C (Supplementary Figures S3C, D, S9A, B). Counting beads were added to the samples after the final washing step.

Samples were acquired on Cytek Aurora and analyzed with FlowJo^TM^ software v10 (BD Life Sciences). Corrected cell counts ${C}_{c}$ were calculated from measured counts ${C}_{m}$ by determining the correction factor ${F}_{c}$

1 $${F}_{c}=\frac{1}{{F}_{o}}\cdot \frac{{B}_{t}}{{B}_{r}}$$

with ${F}_{o}$ the fraction of the organ used in this sample, *B*_*t*_ the total amount of counting beads added and ${B}_{r}$ the amount of beads recovered. For the lymph nodes, the ${F}_{o}$ was 0.5 for the non-ACT cohorts and $\frac{1}{3}$ for the ACT cohorts, meaning the full lymph node was divided between the CD8^+^-specific and pan-immune panel or the CD8^+^-specific, pan-immune and CD90.1^+^CD8^+^-specific panels, respectively (Supplementary Figure S3C, D). In a few of the larger tdLNs at the relapse time point, a lower fraction was used for flow cytometry and the remaining tissue was frozen. The corrected cell counts were calculated according to ${C}_{c}={C}_{m}\cdot {F}_{c}$.

### Multiplex immunofluorescence imaging

For the analysis of multiplex immunofluorescence imaging using the PhenoCycler (formerly CODEX) platform, mouse lymph nodes were fixed in BD Cytofix/Cytoperm at 1:4 dilution in PBS for 4 h at 4 ^∘^C. Tissues were then washed three times in PBS and dehydrated in 15% sucrose solution in PBS for 1 hour at 4 ^∘^C. This was followed by further dehydration in 30% sucrose solution in PBS overnight at 4 ^∘^C. After dehydration, tissues were gently embedded in a cryomold filled with Tissue-Tek OCT (Sakura #4583). Tissues were equilibrated in the Tissue-Tek OCT at room temperature for 20 min and then stored on dry ice for rapid freezing. When completely frozen, tissues were stored at -80 ^∘^C for long-term storage.

For staining, the dehydrated frozen tissues were cryo-sectioned at 5 *μ*M thickness onto 0.1% poly-L-lysine (Sigma-Aldrich #p8920) coated coverslips. Antibody conjugations and tissue staining were performed as previously described^56^. Briefly, the tissue sections were rehydrated in 1x TBS wash buffer with Tween 20 (Thermo Scientific #28360) and stained using the following antibodies against mouse: Tox, FoxP3, Bcl2, Tbet, CD90.1, CD8, CD103, PD1, CD3, Ly6C, ERTR7, Tcf1, Eomes, Ki67, Ly108, Lag3, CD11c, CD45, CD169, CD69, Vimentin, NKp46, CD31, CD21/35, CD11b, CD4, B220, MHCII, F4/80, *α* SMA and Ly6G (Supplementary Figure S3A).

All antibodies were conjugated in-house to oligonucleotide sequences synthesized by biomers.net GmbH. Images were acquired on a PhenoCycler-Fusion (Akoya Biosciences) coupled to a Zeiss Axio Observer 7 inverted microscope through the CODEX Instrument Manager (CIM; Akoya Biosciences) and ZEN (Zeiss) softwares. Every cycle included the acquisition of DAPI, ATTO 550, DY-647P1 and DY-747P1.

#### Multiplex immunofluorescence image processing

Multiplex immunofluorescence images were processed with an in-house pipeline^57^ building on existing methods^58–62^.

Following this, initial phenotyping was done based on automatic cell type clustering using a reference phenotyping tree and the mean fluorescence intensity (MFI) per marker as extracted by in-house code. Clustering was done using Leiden clustering^63^ using the python package scanpy (v1.9.6)^64,65^. Clusters were assigned to predefined cell phenotypes. The plausibility of this assignment was evaluated by inspecting the marker MFI per cluster using the dotplo and UMAP^66^ functionality as implemented by scanpy (Fig. 3D, E).

In cases where the marker intensity profiles of a cluster did not reflect any known phenotype, a subset of the cluster was investigated by inspecting small patches of the image. These patches showed the cell within its direct spatial context, showing the DAPI stain as well as three to five other relevant markers. This visual examination was important to avoid erroneous cell type assignment based on spatial spillover effects or artifacts. After this manual inspection, the cluster was then either assigned to a cell type, discarded as artifact or split further using Leiden clustering. In this way, the dataset was iteratively curated into 22 biologically coherent clusters (Supplementary Figures S3B).

#### Spatial analysis

Nearest neighbor graphs were constructed from each image using the Squidpy spatial_neighbors function (v1.6.0), with a connection threshold of 99.5% of the total distances and 6 nearest neighbors per point^67^. This left the graph with outliers that were disconnected entirely or only with a few other outliers. Outlying points were then filtered out if they contained less than 1% of the points in the main graph.

For lymph node anatomical zone classification, cells were assigned one of twenty neighborhoods using *k*-nearest neighbor clustering. A nearest neighbor count of *k* = 50 was chosen so that assignment was based on a relatively large spatial context, reflecting tissue anatomy. The twenty clusters were manually assigned to one of three tissue regions (T cell zone, B cell follicle and medulla/SCS/interfollicular zone) based on cell type composition and tissue location. Regions were smoothed by evaluating cell alignment with region labels in neighboring cells (Fig. 4A). Smoothing was done with a deterministic Metropolis-type algorithm. Briefly, for each cell with one of three tissue region labels, the *k* = 10 nearest neighbors were determined. Among these neighbors, we determined the most common label ${L}_{most}$ and computed

2 $$p=\frac{\#{L}_{most}}{k}$$

The tissue zone label was deterministically updated according to:

3 $${L}_{new}=\left\{\begin{array}{ll}{L}_{most}, & \text{if } {L}_{orig}\ne {L}_{most}\text{ and } p > 0.5,\\ {L}_{orig}, & \mathrm{otherwise}.\end{array}\right.$$

A threshold of 0.5 was chosen in this three-label setting to enable reassignment of surrounded individual points and a slight edge smoothing, but allow label retention in balanced edge regions. Tissue label reassignment was iterated until effective convergence, defined as a state in which the remaining label changes were exclusively oscillatory. Oscillatory behavior was defined as cells alternating between two labels across successive iterations, such that all changes introduced in iteration *i* − 1 were reversed in iteration *i*. In total, 3.6% of cells that underwent any reassignment remained oscillatory (0.2% of all cells; Supplementary Figure S5A, B), predominantly involving the medulla/SCS/interfollicular zone (Supplementary Figure S5C). Based on this criterion, the maximum number of iterations was set to 25, by which point each image had reached effective convergence (Supplementary Figure S5B).

Cell type abundance was quantified per tissue region. For region proximity analysis, a concave hull was calculated around the T cell zones and distance of cells to the hull border calculated using the SPACEc patch_proximity_analysis function (v0.1.2) using the border_cell_radius method^68^. To assess whether neutrophils outside the T cell zone accumulated preferentially near the zone border, we performed an exploratory distance-to-border analysis using concentric hull-based regions extending 20, 60 and 100 *μ*m from the T cell zone boundary. Cells within and outside each distance-defined region were quantified. The 20 *μ*m distance was selected to represent a near-border compartment corresponding to a few cell diameters, consistent with the spatial scale of direct cell-cell interactions and with proximity thresholds used in related multiplex spatial analyses^69,70^. Larger distances (60 and 100 *μ* m) were included to assess whether any enrichment was restricted to the immediate border region or extended more broadly into the surrounding tissue.

### Flow cytometry-multiplex immunofluorescence imaging comparison

Consensus cell types were defined as cell types present in both modalities. These were grouped by organ and experimental condition. The mean and one standard deviation of the per-sample cell type fractions were determined. Spearman’s rank correlation coefficient was used to determine alignment along the diagonal for these fractions. This nonparametric measure was chosen because it is scale-independent.

### Statistical analysis

Statistical analysis between treatment groups was performed using ANOVA with Tukey’s HSD for family-wise error correction. All data used in these analyses was normally distributed, as tested using the Shapiro-Wilk test. Comparisons within groups between different organs were done using ratio-paired T tests (for normally distributed data) or the Wilcoxon signed-rank test (for non-normally distributed data). Normality was tested using the Shapiro-Wilk test. For exploratory proximity analyses across multiple distance thresholds, P-values were adjusted using the Holm method to control the family-wise error rate. P-values lower than 0.05 were considered statistically significant. P-values were denoted as follows: **** for *p* < 0.0001, *** for 0.0001 ≤ *p* < 0.001, ** for 0.001 ≤ *p* < 0.01, and * for 0.01 ≤ *p* < 0.05. Analysis was done in Python (v3.10) using scipy (v1.15.1), statsmodels (v0.14.4) and scikit_posthocs (v0.11.2).

### Ethics statement

All animal experiments were approved by the local government authorities (Landesamt für Verbraucherschutz und Ernährung (LAVE), North Rhine-Westphalia, Germany) and conducted in accordance with legislation of the national and institutional animal welfare bodies.

## Supplementary information

Supplementary Information

## Data Availability

Data is available in Zenodo at 10.5281/zenodo.18712088.

## Code availability

The original code for this manuscript is available in Zenodo at 10.5281/zenodo.18712088 and on https://github.com/ICB-DCM/ACT_melanoma_neutrophil.
