# Supplementary material for: Longitudinal spatial neutrophil profiling during ACT in murine melanoma reveals distinct lymph node infiltration patterns
Source: NPJ Syst Biol Appl. 2026 Jun 11;12:84. doi: 10.1038/s41540-026-00765-5 (PMC13261029; doi:10.1038/s41540-026-00765-5)
Supplement: Supplementary file 1 — Supplementary Information [file 41540_2026_765_MOESM1_ESM.pdf]

Supplementary materials for the manuscript: Longitudinal spatial neutrophil profiling during ACT in murine melanoma reveals distinct lymph node infiltration patterns

Gemma van der Voort [1,2,3,*], Maike Effern [1,*], Michelle C. R. Yong [1], Lukas Kiwitz [1,3], Roberta Turiello [1], Sonia Leonardelli [1], Susanna Ng [1], Dillon Corvino [1], Tobias Bald [1], Nicole Glodde [1], Kevin Thurley [1,3,†], Jan Hasenauer [2,3,†], Michael Hölzel [1,†]

[1]Institute for Experimental Oncology, University Hospital Bonn, Bonn, Germany

[2]Life and Medical Sciences (LIMES) Institute, University of Bonn, Bonn, Germany

[3]Bonn Center for Mathematical Life Sciences, University of Bonn, Bonn, Germany

[*]These authors contributed equally.

[†]These authors jointly supervised this work. Correspondence: michael.hoelzel@ukbonn.de

Table S1: **Dataset overview.** CODEX: co-detection by imaging, FCM: flow cytometry. The *Mice: total* column counts the total amount of mice per experimental condition. The *Mice: tumor development* column counts the mice that developed a tumor of 9 mm^2 after subcutaneous (s.c.) tumor inoculation. Only these mice were considered for further analysis for all conditions (except Naïve). Columns to the right of the *tumor development* column are a subset of that column. Thus, 8 out of 19 tumor-bearing mice with tumor development were female. Of the 10 mice with tumor development in adoptive T cell therapy (ACT) day 3, flow cytometry data was generated from 6 and co-detection by imaging (CODEX) data from 4 individuals. For some conditions (e.g. Tumor-bearing, Relapse), samples were gathered for CODEX imaging but not processed due to time constraints. For these conditions, the *Mice: FCM* and *Mice: CODEX* columns do not add up to the *Mice: tumor development* column. The final two columns show cell counts per lymph node (section). Since this averages the tumor-draining lymph node (tdLN) and other lymph nodes, the standard deviations are large.

**Treatment**	**Mice: total**	**Mice: tumor development**	**Mice: female**	**Mice: FCM**	**Mice: CODEX**	**FCM: Mean live cell count per LN (±sd)** [10^6]	**CODEX: Mean live cell count per LN section (±sd)** [10^3]
Naïve	24	0	11	8	0	7.04 (±3.06)	–
Tumor-bearing	24	19	8	9	2	5.84 (±5.67)	17.84 (±17.60)
Cy	23	16	5	11	3	3.62 (±5.91)	30.16 (±18.24)
ACT day 3	14	10	5	6	4	7.30 (±10.09)	30.52 (±13.43)
ACT day 7	15	15	5	7	6	14.07 (±18.14)	30.93 (±15.73)
ACT day 14	22	20	4	14	6	17.89 (±29.77)	33.59 (±18.86)
Relapse	24	17	8	14	0	65.22 (±100.89)	–
Untreated	17	13	4	0	0	–	–
ACT day 7 no CpG	6	5	0	5	0	3.83 (±4.06)	–
ACT day 14 no CpG	9	9	0	9	0	19.40 (±29.06)	–
**Total**	178	124	50	83	21	21.92 (±55.19)	30.46 (±16.75)

Table S2: **ACT efficacy overview.** For all treatment groups except Naïve, the number of mice experiencing specific tumor development events is reported. Definitions of tumor development, treatment response, tumor eradication and relapse can be found in the Methods. For per-mouse tumor growth trajectories see **Supplementary Figure S1**.

**Treatment**	**Mice: total**	**Mice: no tumor development**	**Mice: treatment response**	**Mice: tumor eradication**	**Mice: relapse**	**Time to relapse (±sd) [days]**
Tumor-bearing	24	5	0	0	0	–
Cy	23	7	0	0	0	–
ACT day 3	14	4	0	0	0	–
ACT day 7	15	0	2	0	0	–
ACT day 14	22	2	13	0	0	–
Relapse	24	7	16	1	15	18.9 ± 10.7
Untreated	17	4	0	0	0	–
ACT day 7 no CpG	6	1	0	0	0	–
ACT day 14 no CpG	9	0	4	0	0	–
**Total**	154	30	34	1	15	18.9 ± 10.7

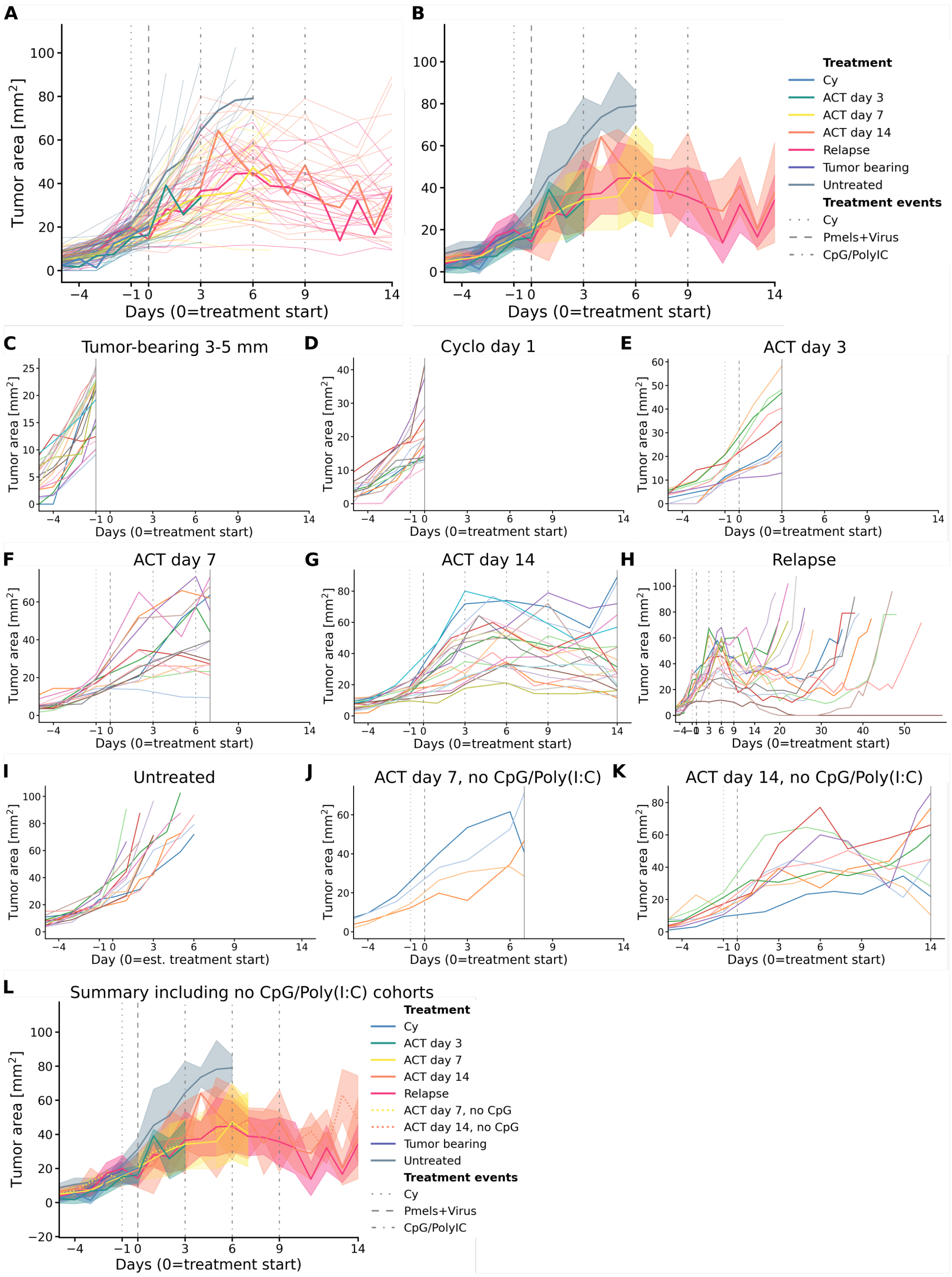

Figure S1: **Tumor growth curves normalized by treatment starting date.** **(A, B)** Individual and averaged trajectories of all non-perturbation conditions. The shaded area in **(B)** indicates ±1 standard deviation from the population mean. **(C-K)** Individual growth trajectories per mouse per condition. The solid line indicates the sacrifice event. **(I)** Treatment start estimated as the day after tumor reached 16 mm^2, the mean intended size. **(L)** Averaged trajectories including the conditions without CpG/Polyinosinic:polycytidylic acid (CpG/Poly(I:C)) stimulation. The shaded area indicates ±1 standard deviation from the population mean.

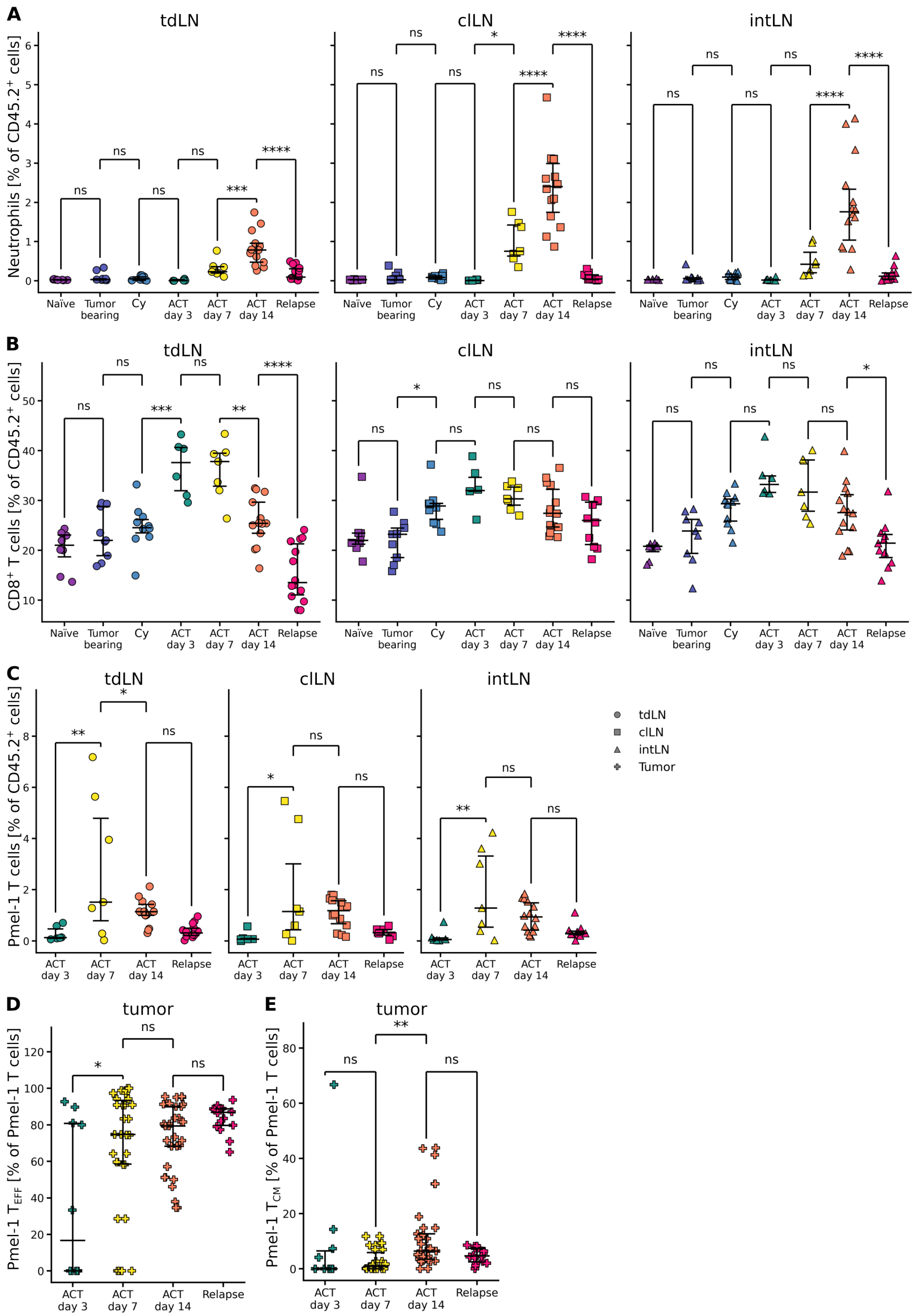

Figure S2: **Abundances of major cell types in the lymph nodes and tumor between conditions. (A-C)** Neutrophils, $CD8^+$ T cells and Pmel-1 T cells in the tumor draining (left), contra-lateral (middle) and intermediate (right) lymph nodes. **(D, E)** Relative abundances of effector $CD8^+$ T cell (T_{EFF}) and central memory $CD8^+$ T cell (T_{CM}) in the tumor. Unpaired tests **(A-E)** performed using ANOVA with Tukey's HSD for family-wise error correction. ****: $p < 0.0001$, ***: $p < 0.001$, **: $p < 0.01$, *: $p < 0.05$ and ns: $p \geq 0.05$. All data was obtained using flow cytometry.

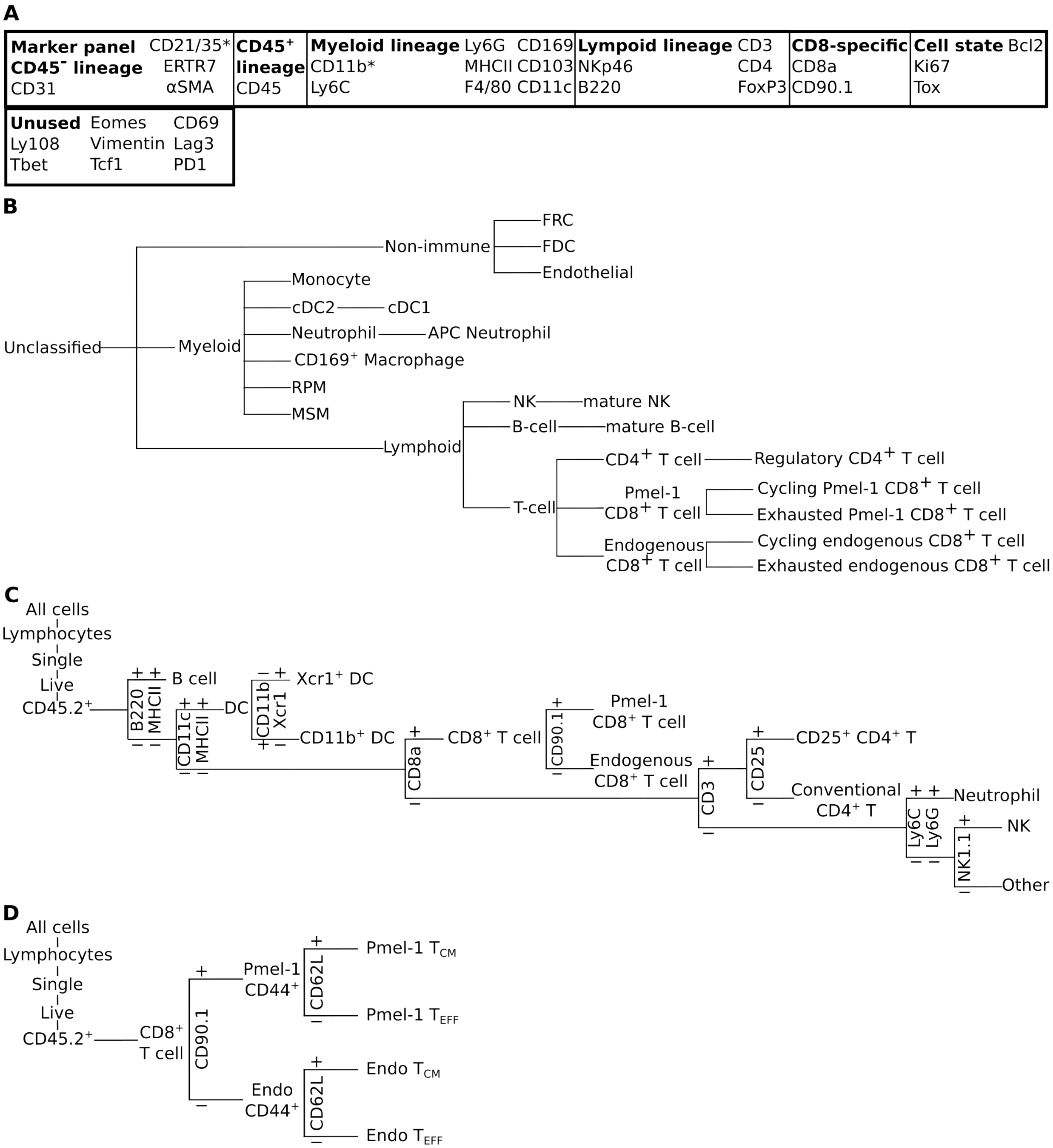

Figure S3: **Overview of phenotyping strategies. (A)** Multiplex immunofluorescence imaging marker overview. 22 markers where used for phenotyping. A * indicates a marker was used for phenotyping more than one cell type. **(B)** Multiplex immunofluorescence imaging phenotyping tree. Exhausted Pmel-1 $CD8^+$ T cells were not found. **(C)** Flow cytometry pan-immune panel. **(D)** Flow cytometry $CD8^+$-specific panel.

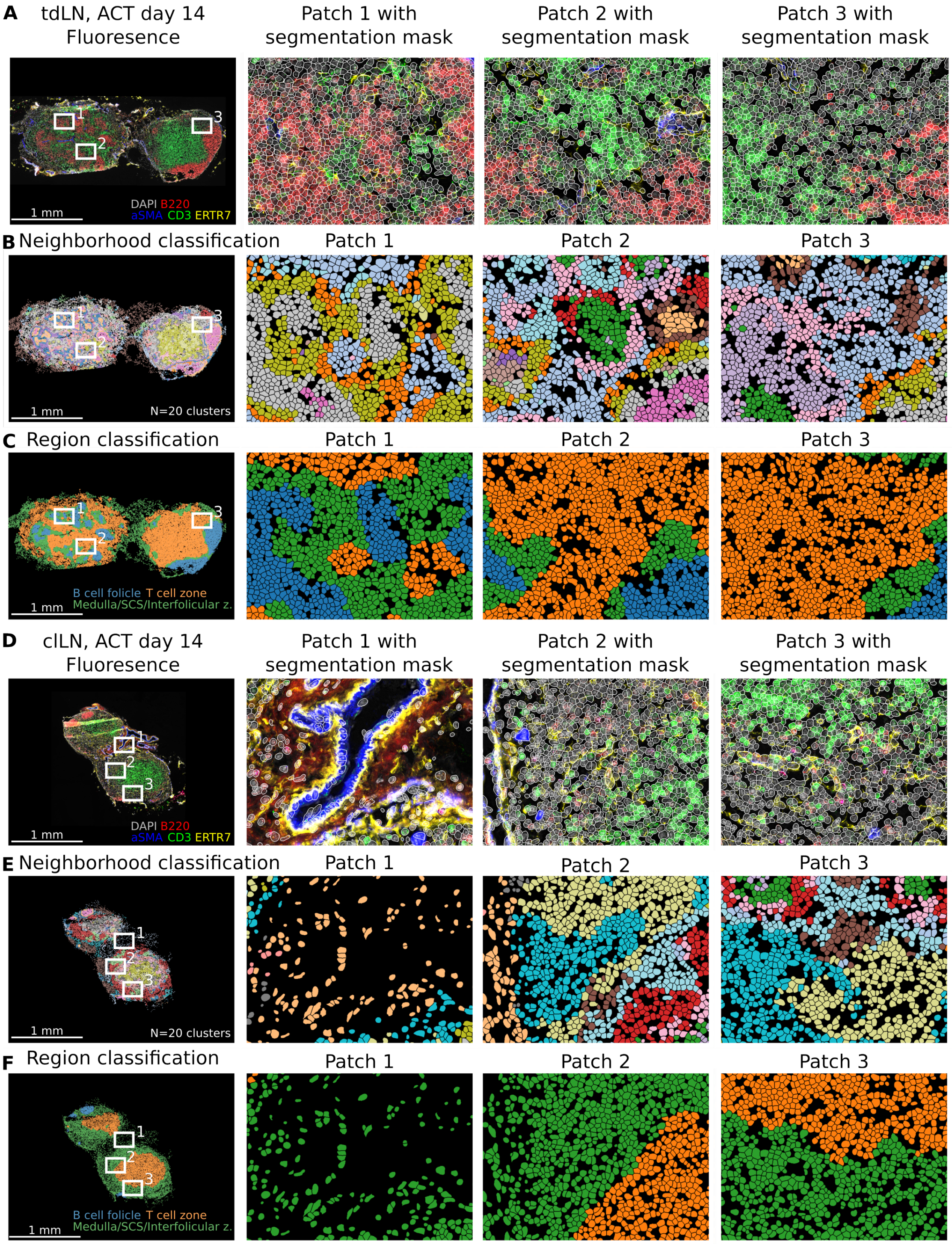

Figure S4: **Additional illustration of segmentation, niche and region classification in multiplex immunofluorescence imaging analysis (A, D)** tdLN **(A)** and contralateral lymph node (clLN) **(D)** at ACT day 14 with segmentation masks for three image patches. Selected markers: DAPI (grey), B220 (red), aSMA (blue), CD3 (green), ERTR7 (yellow). **(B, E)** Lymph node and zooms from **(A, D)** with neighborhood classification based on $k = 50$ nearest neighbors and $n = 20$ clusters. **(C, F)** Lymph node and zooms from **(A, D)** with smoothed region classification. B cell follicle (blue), T cell zone (orange) and Medulla/subcapsular sinus (SCS)/Interfollicular zone (green).

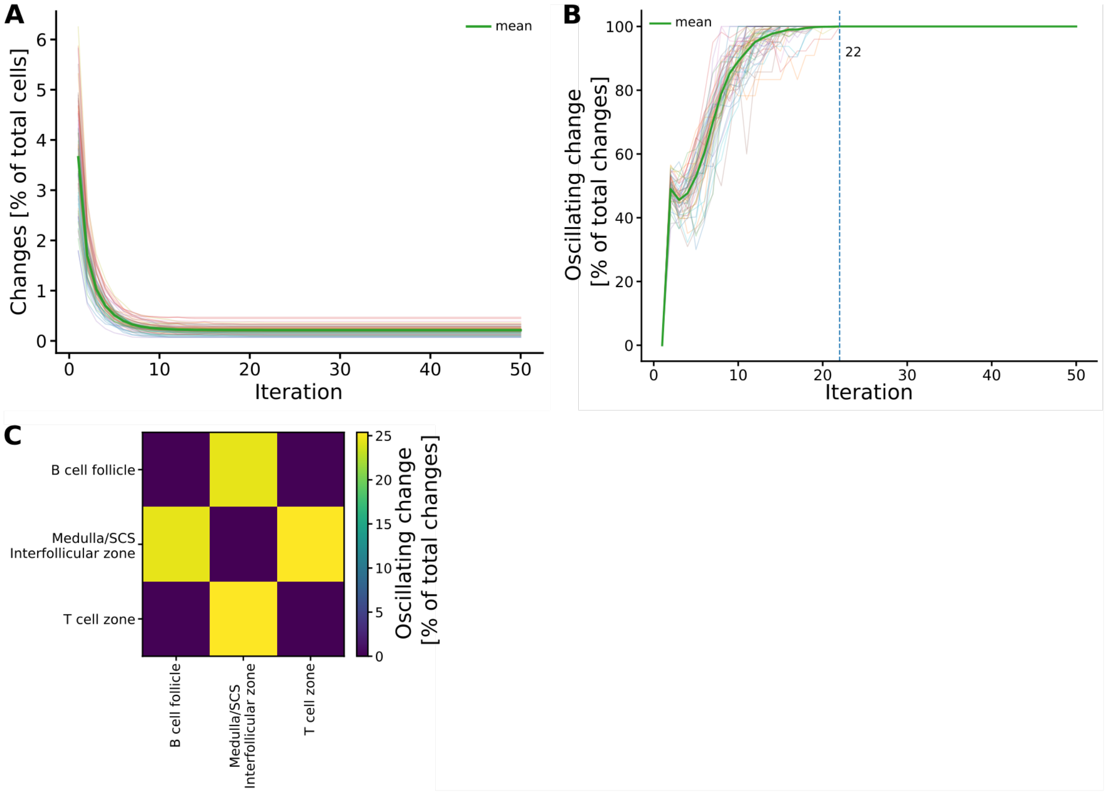

Figure S5: **Sensitivity analysis of region smoothing algorithm**. **(A)** Number of label reassignments per iteration, per image. **(B)** Number of oscillating changes per iteration, per image. **(C)** Heatmap of pairwise oscillatory label transitions between tissue regions, indicating the percentage of total oscillating changes attributed to each region pair.

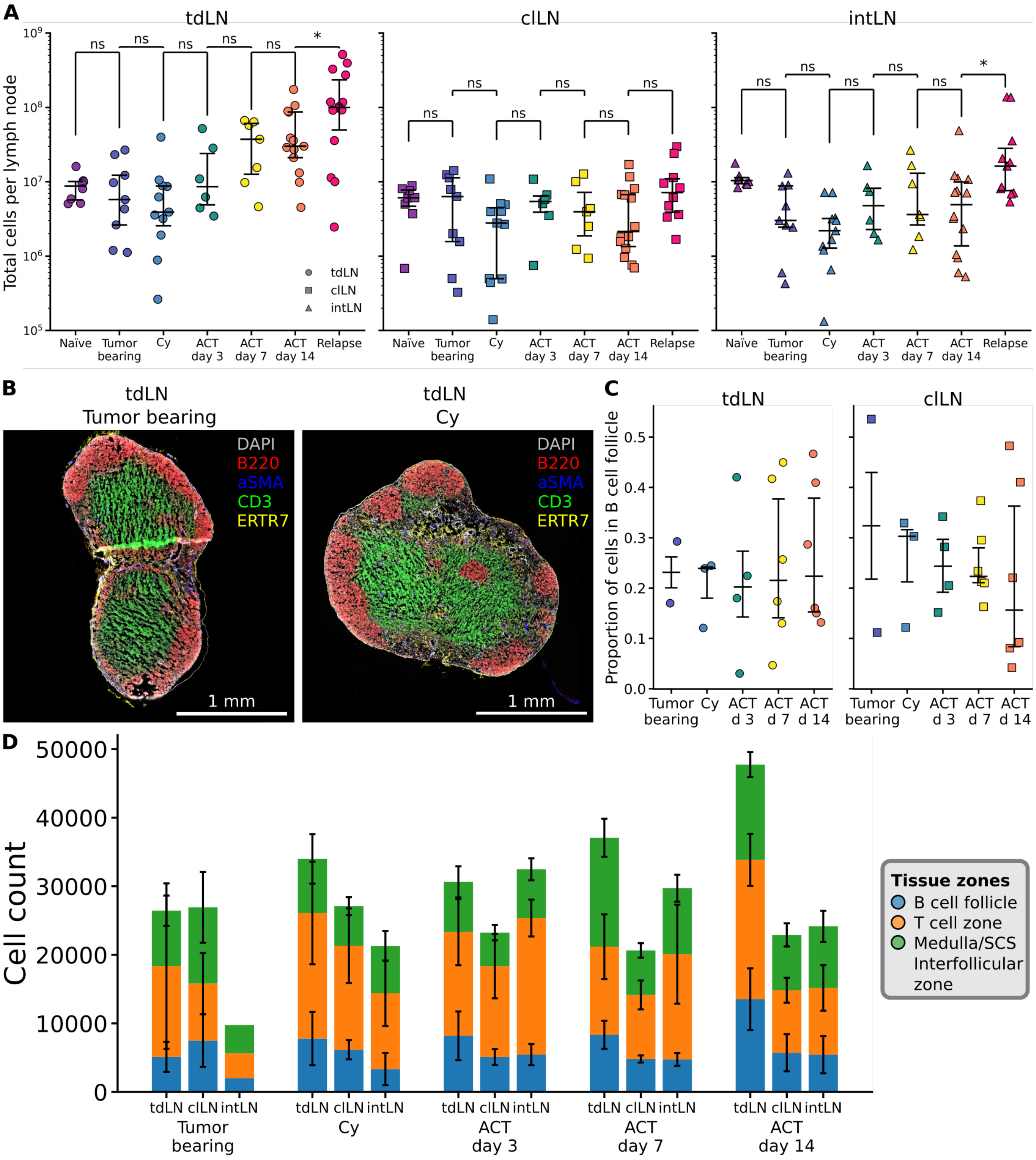

Figure S6: **Size differences, architectural similarities and compositional changes in the lymph nodes.** **(A)** Total live cell counts per lymph node in flow cytometry data, compared between conditions. **(B)** Representative tdLN lymph nodes in the tumor bearing and cyclophosphamide (Cy) conditions. Selected markers: DAPI (grey), B220 (red), aSMA (blue), CD3 (green), ERTR7 (yellow). **(C)** Relative size of the B cell follicle compartment in the tdLN and clLN. **(D)** Averaged sizes of the lymph nodes in the imaging data, split by compartment. Error bars indicate standard error of the mean. Unpaired tests **(A, C)** performed using ANOVA with Tukey's HSD for family-wise error correction. ****: $p < 0.0001$, ***: $p < 0.001$, **: $p < 0.01$, *: $p < 0.05$ and ns: $p \geq 0.05$. Non-significant associations in **(C)** are not shown.

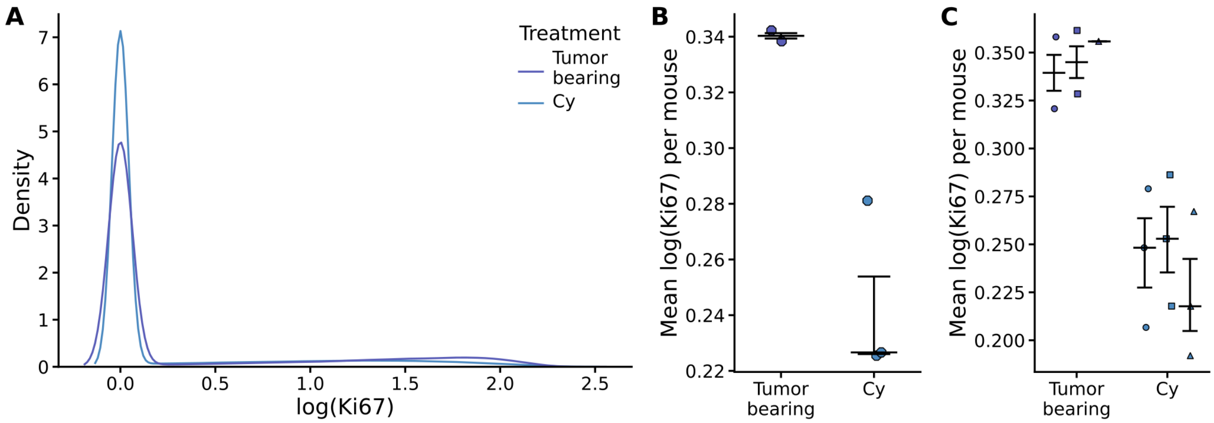

Figure S7: **Ki67 expression in imaging data pre- and post-Cy treatment (A)** Distribution of log-transformed Ki67 intensity in tumor-bearing and Cy conditions. **(B, C)** Mean log-transformed Ki67 intensity per mouse **(B)** and per lymph node **(C)** for the tumor-bearing and Cy conditions.

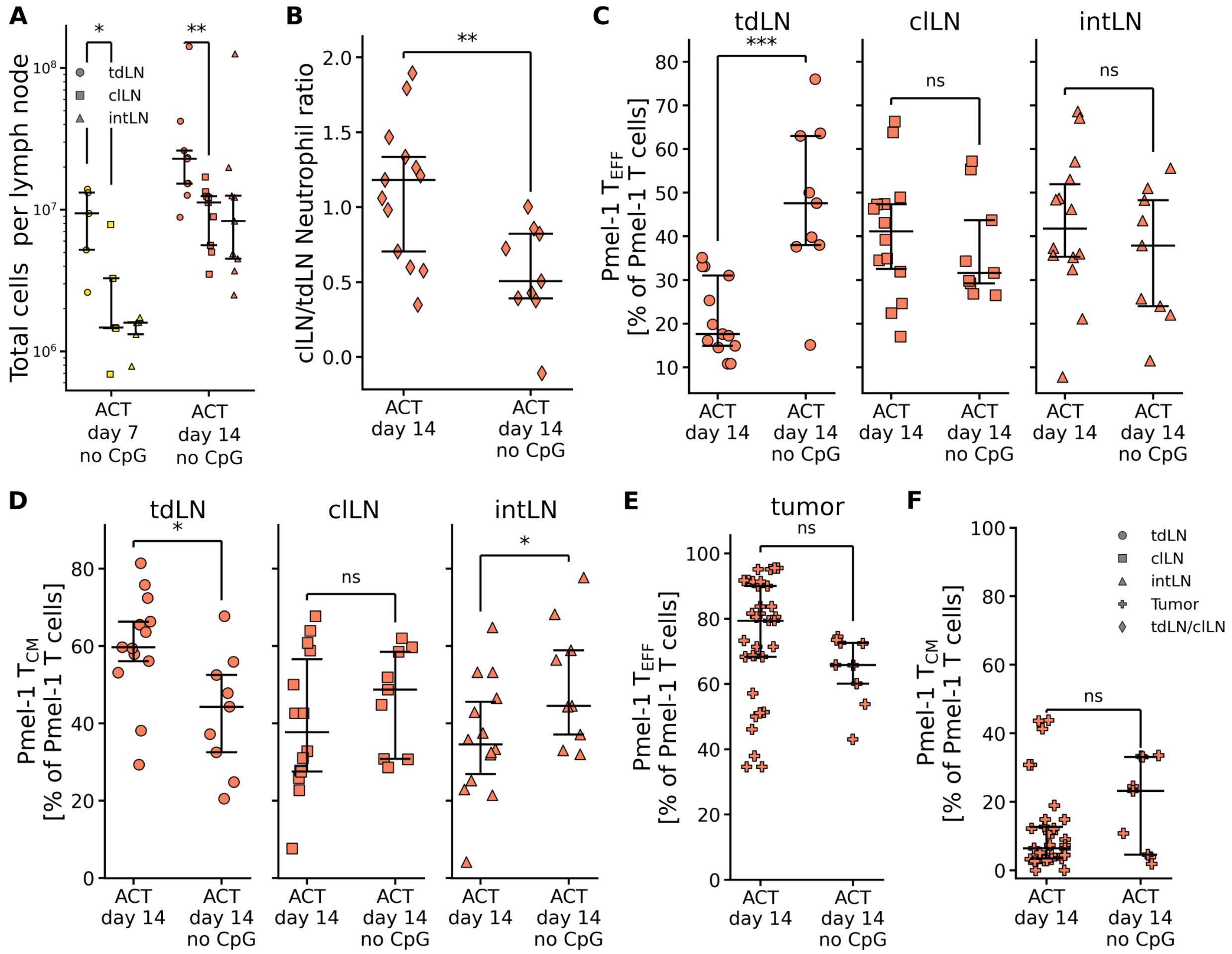

Figure S8: **Additional comparisons on the omission of innate immune stimulation in ACT day 7 and 14.** **(A)** Lymph node sizes with and without CpG/Poly(I:C) treatment on day 7 and 14. **(B)** The log $\frac{\text{clLN}}{\text{tdLN}}$ of neutrophils as a percentage of $CD45^+$ cells with and without CpG/Poly(I:C) treatment at day 14. **(C, D)** T_{EFF} and T_{CM} with and without CpG/Poly(I:C) treatment at day 14, per lymph node. **(E, F)** T_{EFF} and T_{CM} in the tumor. Paired tests **(A)** performed using a ratio-paired T test. Unpaired tests **(B-E)** performed using ANOVA with Tukey's HSD for family-wise error correction. ****: $p < 0.0001$, ***: $p < 0.001$, **: $p < 0.01$, *: $p < 0.05$ and ns: $p \geq 0.05$. All data was obtained using flow cytometry.

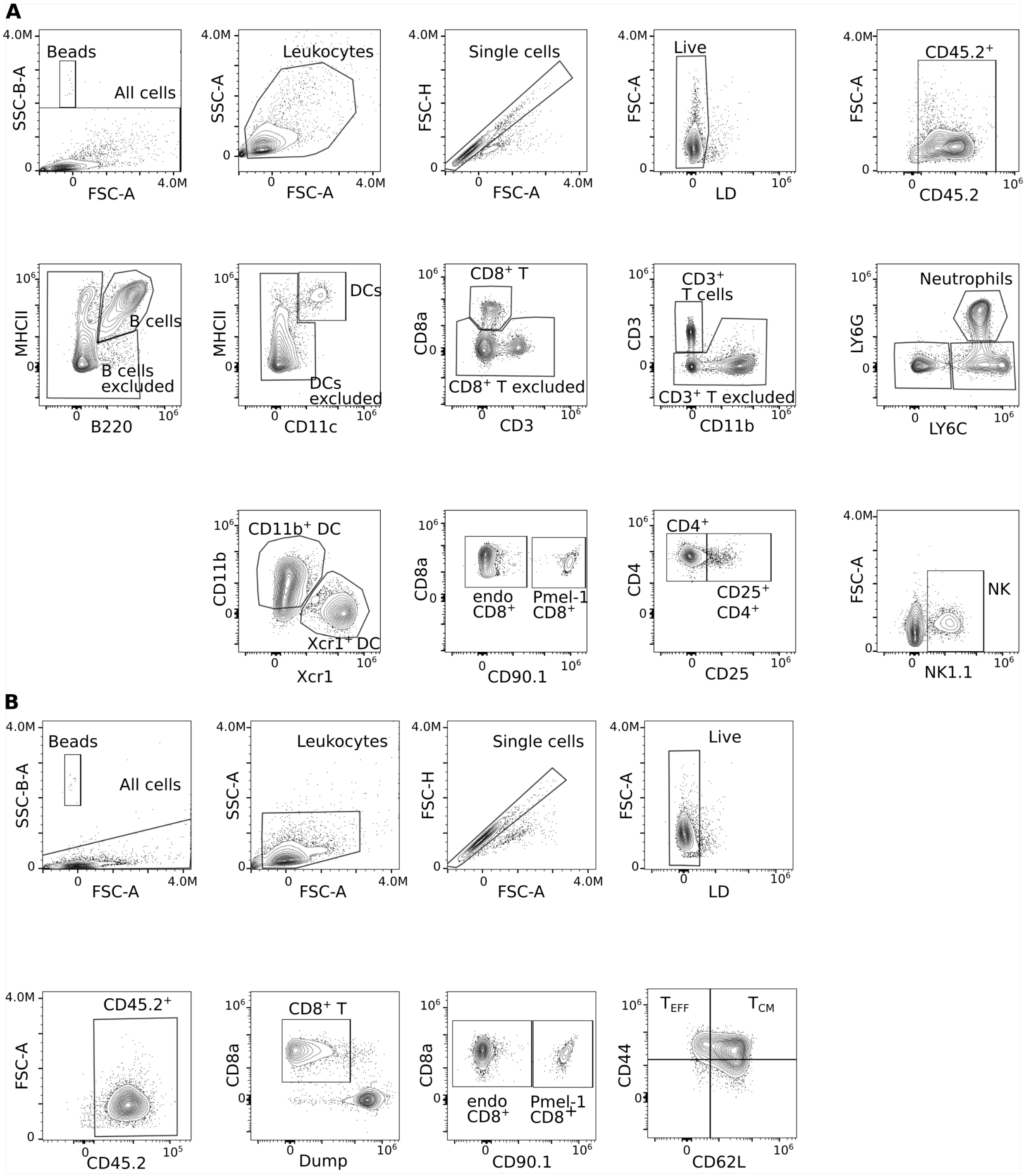

Figure S9: **Details of flow cytometry gating strategy.** **(A)** pan-immune panel **(B)** CD8$^+$-specific panel.
